# Oral administration of a recombinant modified RBD antigen of SARS-CoV-2 as a possible immunostimulant for the care of COVID-19

**DOI:** 10.1186/s12934-024-02320-5

**Published:** 2024-02-06

**Authors:** Norma A. Valdez‑Cruz, Diego Rosiles-Becerril, Constanza E. Martínez-Olivares, Enrique García‑Hernández, Laura Cobos-Marín, Daniel Garzón, Francisco E. López-Salas, Guadalupe Zavala, Axel Luviano, Alejandro Olvera, Alejandro Alagón, Octavio T. Ramírez, Mauricio A. Trujillo‑Roldán

**Affiliations:** 1 Departamento de Biología Molecular y Biotecnología, Instituto de Investigaciones Biomédicas, Universidad Nacional Autónoma de México, Cd. Universitaria, Coyoacán, Ciudad de Mexico, México. AP. 70228, CP. 04510 México, D.F, Mexico; 2 Centro de Nanociencias y Nanotecnología, Universidad Nacional Autónoma de México, Km 107 Carretera, 22860 Tijuana-Ensenada, Baja California Mexico; 3 Instituto de Química, Universidad Nacional Autónoma de México, Ciudad Universitaria, 04510 Ciudad de México, Mexico; 4 Departamento de Microbiología e Inmunología, Facultad de Medicina Veterinaria y Zootecnia, Universidad Nacional Autónoma de México, Ciudad Universitaria, 04510 Ciudad de México, Mexico; 5 Unidad de Modelos Biológicos, Instituto de Investigaciones Biomédicas, Universidad Nacional Autónoma de México, Cd. Universitaria, Coyoacán, Ciudad de Mexico, Mexico. AP. 70228, CP. 04510 México, D.F, Mexico; 6 Unidad de Microscopia Electrónica, Instituto de Biotecnología, Universidad Nacional Autónoma de México, Cuernavaca, Mor Mexico; 7 Departamento de Genética del Desarrollo y Fisiologia Molecular, Instituto de Biotecnología, Universidad Nacional Autónoma de México, Cuernavaca, Mor Mexico; 8 Departamento de Biología Molecular y Bioprocesos, Instituto de Biotecnología, Universidad Nacional Autónoma de México, 62210 Cuernavaca, Mor Mexico

## Abstract

**Background:**

Developing effective vaccines against SARS-CoV-2 that consider manufacturing limitations, equitable access, and acceptance is necessary for developing platforms to produce antigens that can be efficiently presented for generating neutralizing antibodies and as a model for new vaccines.

**Results:**

This work presents the development of an applicable technology through the oral administration of the SARS-CoV-2 RBD antigen fused with a peptide to improve its antigenic presentation. We focused on the development and production of the recombinant receptor binding domain (RBD) produced in *E. coli* modified with the addition of amino acids extension designed to improve antigen presentation. The production was carried out in shake flask and bioreactor cultures, obtaining around 200 mg/L of the antigen. The peptide-fused RBD and peptide-free RBD proteins were characterized and compared using SDS-PAGE gel, high-performance chromatography, and circular dichroism. The peptide-fused RBD was formulated in an oil-in-water emulsion for oral mice immunization. The peptide-fused RBD, compared to RBD, induced robust IgG production in mice, capable of recognizing the recombinant RBD in Enzyme-linked immunosorbent assays. In addition, the peptide-fused RBD generated neutralizing antibodies in the sera of the dosed mice. The formulation showed no reactive episodes and no changes in temperature or vomiting.

**Conclusions:**

Our study demonstrated the effectiveness of the designed peptide added to the RBD to improve antigen immunostimulation by oral administration.

**Graphical Abstract:**

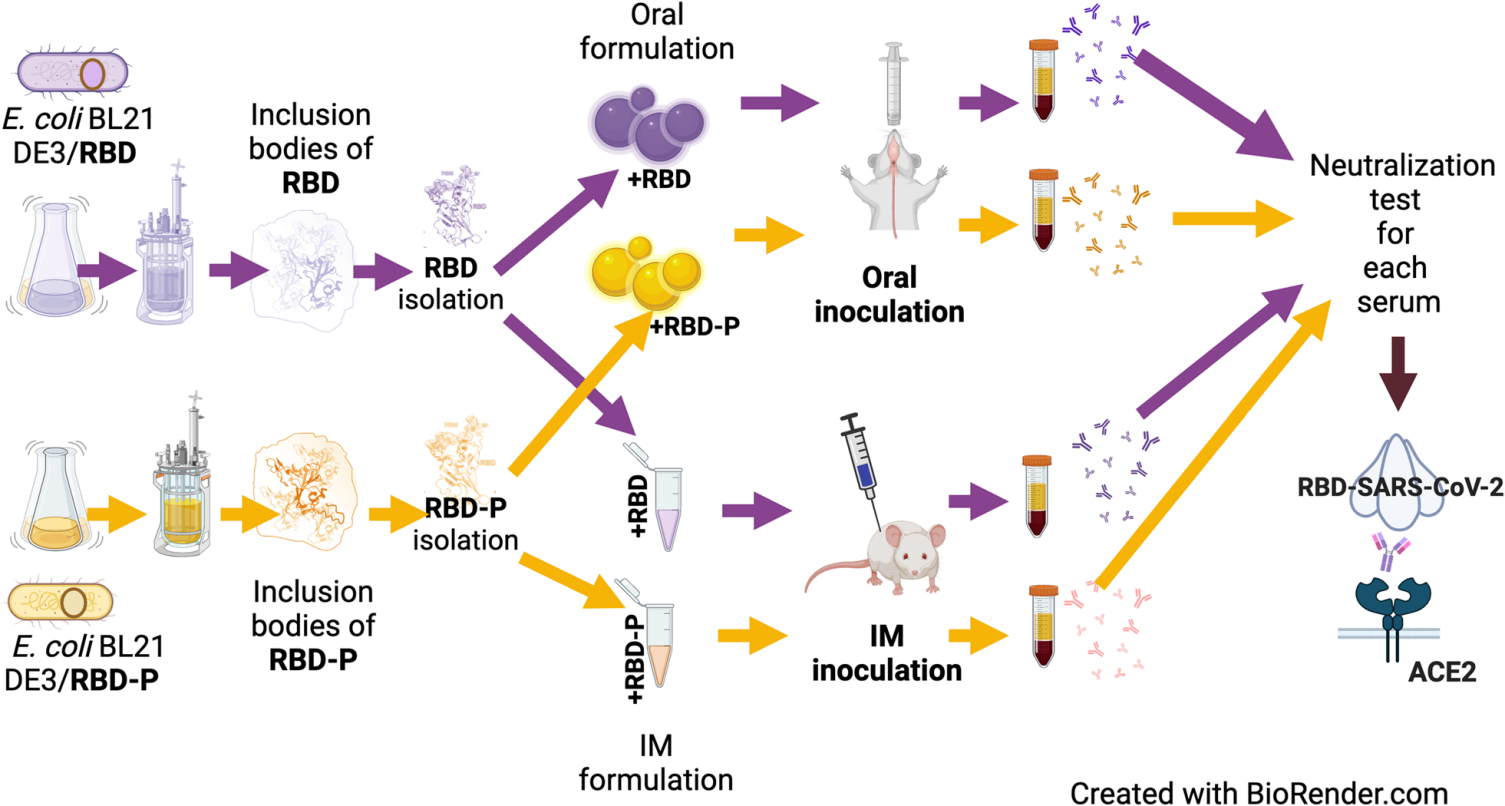

**Supplementary Information:**

The online version contains supplementary material available at 10.1186/s12934-024-02320-5.

## Introduction

COVID-19 is a severe acute respiratory syndrome caused by coronavirus 2 (SARS-CoV-2), affecting public health and the economy for at least three years. The World Health Organization (WHO) described the SARS-CoV-2 virus as having affected more than 671 million people, causing almost 7 million deaths, by September 2023 [1]. In general, the symptoms of COVID-19 are fever, cough, and shortness of breath, although they can vary depending on the variant [2, 3]. Shortly after the disease spread rapidly worldwide, the first recombinant vaccines and antibodies were generated and authorized for emergency use. The fast-developed vaccines based on DNA plasmids, messenger RNA, recombinant proteins, and nanoparticles have reached more than 13.3 billion doses administrated in around 2 years [4–6]. However, research is still needed to develop secure and effective vaccines and corresponding efficient bioprocesses for their production, coupled with translational and clinical studies for anticipating possible new pandemics.

By March 2023, more than 380 candidate vaccines were in development or approved for clinical evaluation (183 vaccines in clinical development and 199 vaccine candidates in preclinical development) [7]. Most of the approved emergency vaccines were designed based on the viral sequence identified in Wuhan, China, incorporating strategies such as adenoviral vector (ChAdOx1 nCoV-19, AstraZeneca), mRNA [BNT162b2 (BNT), Pfizer-BioNTech, and mRNA-1273 (m1273), Moderna], and recombinant proteins incorporated in nanoparticles [NVX-CoV2373 (NVX), Novavax]. Some vaccines still show effectiveness on different variants of the virus. However, a decrease in effectiveness has been detected against some variants such as B.1.1.248 and B.1.351 [8] and omicron [9–11].

The appearance of new variants of concern (VOC) causes changes in the effectiveness of the vaccines in use [12–16]. For example, vaccine efficacy against the B.1.351 variant was reduced by 30 to 40% for lNVX-CoV2373 (Novavax), JNJ-78436735 (Johnson & Johnson), and Oxford-AstraZeneca ChAdOx1 [17]. While the efficacy of the lNVX-CoV2373 (Novavax) vaccine was reduced from 96% with Wuhan to 51% against the B.1.351 variant [18], and the Oxford-AstraZeneca ChAdOx1 vaccine decreased from 62 to 10% [17] against the same variant. Accordingly, better vaccines that can control future SARS viruses and vaccine boosters with better effectiveness are needed. Therefore, the development of specific, effective, and safe vaccines and evaluation of their administration routes are necessary to reach the long-term effectiveness of vaccines that could be administered annually according to seasonal variations [19, 20].

The SARS-CoV-2 is a positive-sense, single-stranded RNA virus [21], composed of the main structural proteins Spike (S), Membrane (M), Envelope (E), and Nucleocapsid (N). The glycoprotein S (180-kDa) is a homotrimer that protrudes from the viral surface and mediates the entry of the coronavirus into host cells through the receptor-binding domain (RBD), which binds the human angiotensin-converting enzyme 2 (hACE2) receptor [22, 23]. Each S monomer comprises the subunits S1 and S2 connected by a furin cleavage sequence [24, 25], and the RBD is localized in S1. Many anti-SARS-CoV2 vaccines have been directed towards the RBD and the N-terminal domain (NTD), described as preferentially antigenic sites, consistent with the development of neutralizing antibodies by COVID-19 patients [2, 26]. RBD (residues 319–541) composed of five-stranded antiparallel β-Sheets (β1–4 and β7) linked to three helices (α1–α3) by loops, contains the receptor binding motif (RBM) that interacts with the segments 446–505 of the hACE2 [2, 27].

The SARS-CoV-2 infection causes local immunity involving immunoglobulins, such as IgA and IgG, and stimulates cells that can secrete antibodies in the mucosa of the respiratory tract, nose, lungs, and others [28]. Since RBD is a strategic antigenic site, different unglycosylated and soluble receptor binding domains have been produced in *E. coli*. The different RBD has been formulated with a variety of adjuvants, which, when inoculated intramuscularly or subcutaneously, usually induce polyclonal antibodies that evoke a strong immune response and that inhibit the association of the RBD with ACE2, producing, in some cases, neutralizing antibodies [29, 30]. Usually, oral vaccines are not invasive, safe, and can generate immunity against infectious diseases [31–33]. Oral attenuated vaccines often lead to long-term mucosal and systemic immunity due to the intensified immunity of T-cells at the intestinal epithelial, the inflammatory or anti-inflammatory cytokines, and the elicitation of systemic neutralizing antibodies [32, 34–39]. An essential advantage of oral vaccines is their stability, simplifying logistics and storage conditions [40, 41]. Oral vaccine approaches to treat influenza, dengue, tetanus, diphtheria, and MERS-CoV have been published [42–46]. Until now, oral administration requires more studies for its approval because it usually presents a suboptimal induction of cellular immune responses and neutralizing antibodies.

Nowadays, at least six recombinant oral vaccines against SARS-CoV-2 prototypes have been reported. One system expressed the full-length spike protein receptor (RBD) of SARS-CoV-2 on the surface of *Saccharomyces cerevisiae*, which, after oral administration without adjuvant to mice, produced significant humoral and mucosal responses [47]. Three prototypes expressed SARS-CoV-2 regions in *Lactobacillus* [41, 48, 49]. Particularly, the epitopes expressed on the surface of *Lactiplantibacillus plantarum,* when administered orally, caused immunoregulatory effects, suggesting its ability to induce humoral and mucosal immune responses [41]. An adenovirus-based vaccine containing S and N proteins, administered orally, could confer protection in Male Syrian Hamsters against SARS-CoV-2 [50]. Furthermore, an orally administered SARS-CoV-2 subunit-based vaccine combined with the adjuvant non-toxic B subunit of the heat-labile enterotoxin (LT) of *E. coli* induced systemic neutralizing by IgG and cellular immune responses in mice [51].

The present study took advantage of a strategy that has effectively improved the presentation of some antigens. Here, we engineered a non-glycosylated form of SARS-CoV-2 RBD fused to a 40-residue peptide to improve the protein presentation through oral administration. This strategy resulted in the generation of antibodies capable of neutralizing the RBD interaction with hACE2. We developed an orally administrated oil-in-water emulsion containing the designed fused RBD antigen produced in *E. coli* in a methodology capable of being produced on a large scale. This peptide-fused RBD immunostimulates mice, generating neutralizing antibodies after administration without toxicity, compared with the unglycosylated RBD lacking the peptide tag. Demonstrating that RBD fused protein in oil-in-water emulsions is a powerful biotechnological strategy for improving RBD antigen presentation orally, allowing the production of binding and neutralizing IgG in immunized mice.

## Materials and methods

### Generation of the RBD proteins of the Wuhan and Omicron BA.1 variant belonging to the SARS-CoV-2 virus

We designed a peptide of 40 amino acids (40AV, unpublished) to be fused to the RBD domain of SARS-CoV-2 (named RBD-P) to improve the antigen presentation through an oil-in-water emulsion that could be administered orally and compared with the RBD domain without any tag (named RBD). The plasmids encoding the RBD with peptide 40AV (717 bp) and without peptide (597 bp) genes were synthesized by GenScript Biotech Corporation (USA) and cloned in the plasmid pET15b (Novagen, USA). The RBD sequence (amino acid 330–525) was based on the sequence of the S gene of SARS-CoV-2 WA1/2020 (GenBank: MN908947). Constructed plasmids were purified and used to transform *E. coli* TOP10 and subsequently to transform *E. coli* BL21 (DE3) production cells. Confirmation of each construction was determined by enzyme restriction analysis.

RBD from Omicron BA.1 was produced and named RBD-o. The Omicron BA.1 coding gene was obtained in the vector pcDNA3.1 (donated by Dr. Jesús Hernández; Centro de Investigación en Alimentación y Desarrollo, Sonora, México). The plasmid was used as templates to amplify the coding sequence, using primers (5ʹ-GGATCCCAACCTACAGAGAGCATCGTGCGGTTC-3ʹ), (5ʹ-AAGCTTCTAGAAGTTCAC GCACTTGTTCTTCAC-3ʹ). The product was cloned in the TOPO 2.1 (InvitrogenⓇ) plasmid and amplified in Chemocompetent XL1-Blue cells [52]. Region digested with BamHI and Hind III was subcloned into a pQE30 vector (Qiagen, Germany) and used to transform *E. coli* Shuffle strain (New England Biolabs, USA). The RBD-o produced contains a 6X-His tag. The DNA was sequenced in the Sequencing Unit at the Institute of Biotechnology, UNAM (Cuernavaca, Mexico).

### Shake flasks and bioreactor production of RBD and RBD fused to the peptide (RBD-P)

The Luria–Bertani (LB) medium was used to grow the *E. coli* transformants. The inoculum was carried out in 250 mL conventional Erlenmeyer shake flasks containing 50 mL of medium (Duran Erlenmeyer flask, USA). The inoculum was grown at 37 °C and 200 rpm for 12 h (orbital shaking diameter of 25 mm). Bioreactors were inoculated from shake flasks cultures to an optical density (OD_600nm_) of 0.1 A.U. (Spectronic Genesys 20, Thermo, USA) and kept at 37 °C, with air injection at 1 volume of air per volume of culture medium (vvm). The dissolved oxygen tension (DOT) was controlled at 30% through an agitation cascade via a proportional-integral-derivative (PID) strategy [53]. Production cultures were performed in triplicate in a 1.2 L nominal volume Applikon Bioreactor with 0.8 L of working volume at pH 7.5, controlled by NaOH or HCl (2.5 N) addition. Bioreactors were equipped with temperature, pH, and DOT AppliSens sensors connected to the ADI-1010 biocontroller (Getinge—Applikon Biotechnology, The Netherlands). Recombinant RBD-P and RBD expression was induced by adding isopropyl-β-d-1-thiogalactopyranoside (IPTG, 0.1 mM, Merck-Sigma-Aldrich, USA) after 6 h of culture. The composition of the mineral media used was described by Restrepo-Pineda et al. [54]. The growth of *E. coli* cultures was followed by optical density and converted to dry cell weight (DCW) by a linear-correlation standard curve, where 1 A.U. was equivalent to 0.35 ± 0.02 (g DCW)/L. Culture samples were centrifuged at 7000×*g* for 10 min and then filtered (0.2 μm mixed cellulose ester membrane filter, Merck-Millipore, USA). Glucose was measured in the supernatant using the Y15 automatized analyzer (Biosystems, Barcelona, Spain).

RBD-o clones were cultured in shake flasks of 1 L with 200 mL of LB in the presence of 80 µg/mL of ampicillin at 30 °C and 140 rpm (Duran Erlenmeyer flask, USA, orbital shaking diameter of 25 mm). The recombinant RBD-o production was induced with 0.2 mM IPTG after 10 h of culture. These cultures were harvested at 11,300×*g* in a centrifuge (Beckman J2-21, USA) for 10 min at 8 °C.

### Recovery and quantification of total and insoluble protein

To quantify total cellular protein, biomass was centrifuged at 8000×*g* for 10 min, separated, and suspended in lysis buffer (50 mM Tris–HCl, 1 mM EDTA, 100 mM NaCl, pH 7.5) with 0.1 mM phenylmethylsulfonyl fluoride (PMSF). The biomass suspension was sonicated on a SoniPrep150 (Sanyo-Gallen-Kamp, United Kingdom) using an amplitude of 10 μm and 8 steps of 30 s with intervals of 30 s at 4 °C. Total cellular protein was treated with solubilization buffer IEF (7 M urea, 2 M thiourea, 2% CHAPS w/v, and 40 mM DTT; all from Merck-Sigma-Aldrich, USA) for 3 h for solubilization. Bradford method (Bio-Rad Protein Assay Kit II, Bio-Rad, USA) was used to determine protein concentration. A calibration curve was constructed using bovine serum albumin (BSA, GE Healthcare Bio-Sciences, USA) as standard, and optical density at 600 nm was measured in a 96-well microtiter plate reader (Stat Fax® 4200, Awareness Technology, Inc., USA). Samples and standards were evaluated in triplicate. The total cell protein pattern, solubilized with IEF, was observed by gel electrophoresis (SDS-PAGE) in a 12% gel stained with Coomassie Brilliant Blue G-250 (Merck-Sigma-Aldrich, USA), using the Image-LabTM software on a Gel DocTM EZ System (Bio-Rad, USA). Soluble protein was obtained directly from cell sonication, and insoluble protein was recovered by centrifugation. The pellets were suspended in a lysis solution (Tris–HCl 50 mM, EDTA 1 mM, NaCl 100 mM, pH 7.5 with 0.1 mM Phenylmethyl sulfonyl fluoride, Merck-Sigma-Aldrich, USA). The precipitates were washed in 1% (v/v) Nonidet-P40 (Merck-Sigma-Aldrich, USA), and the samples were shaken. The precipitates, containing the inclusion bodies (IBs) were washed seven times with low-conductivity deionized water to remove traces of detergent and DNA. The percentage of recombinant RBD and RBD-P obtained in IBs was estimated from three biological samples by densitometry on a SDS-PAGE gel at 12%, preceded by an IBs solubilization step using IEF for 8 h, using Image-Lab™ software on a Gel Doc™ EZ System (Bio-Rad, USA). Soluble and insoluble protein concentration was determined by the Bradford method in the 96-well microplate format using Dye Reagent Concentrate (Bio-Rad, USA), and BSA (GE Healthcare Bio-Sciences, USA) was used as standard.

### Recombinant RBD purification

Recombinant RBD and RBD-P in IBs obtained from bioreactor cultures of *E. coli* BL21 (DE3) were subjected to solubilization in denaturing solution for 8 h, for subsequent separation by gel chromatography and electroelution, and dialysis in Milli-Q water filtered by 0.22 μm (Merck-Millipore, USA). Quantification was performed by densitometry on SDS-PAGE using Image-Lab™ and Gel Doc™ EZ Imager software (Bio-Rad, Hercules, CA, USA). The purification of RBD and RBD-P was performed by reverse-phase high-performance liquid chromatography (RP-HPLC, Shimadzu, Kyoto, Japan), using a PROTO300 Semi-Prep C4 column (10 μm, 250 × 10 mm) and the Xbridge Protein BEH C4 column (300 Å, 3.5 mm, 4.6 mm × 150 mm, Waters, USA). Recombinant proteins were eluted with a 0% to 60% acetonitrile gradient. Eluted proteins were identified at 220 nm.

RBD-o cell pellets were lysed, and IBs were obtained by centrifugation (11,300×*g*) and solubilized by 12 h at 20 °C under 2% *N*-lauryl sarcosine in 40 mM Tris–HCl buffer (pH 8). The solutions were centrifuged at 20,400×*g* for 20 min, and the supernatants were dialyzed (6–8 kDa MWCO Spectra Por membranes, Repligen, USA) with ten volumes of phosphate buffered saline solution (PBS) pH 7.2 by 96 h. RBD-o was purified in an agarose-NiNTA column (NovagenⓇ, Germany), eluted with 250 mM imidazole, and dialyzed against PBS.

### TEM analysis of inclusion bodies and droplets of oil in water emulsion of RBD-P and RBD

The morphology and size of the IBs inside cells were analyzed under transmission electron microscopy. Cell samples were taken after 5 h of induction. Briefly, samples were washed three times with 0.16 M sodium cacodylate buffer at pH 7.2 at 4 °C, fixed with 4% paraformaldehyde and 2.5% glutaraldehyde in sodium cacodylate buffer pH 7.4 by 2 h at 4 °C. Samples were post-fixed with 1% osmium tetraoxide for 90 min at 4 °C, and were rinsed twice in chilled buffer and six times in cold distilled water. Then, samples were dehydrated in ethanol series and embedded in Epon/Araldita [55]. Uranyl acetate and lead in citrate were used to stain thin sections and observed with a ZEISS Libra 120 plus electron microscope [56]. At least 100 cells were analyzed for each sample. The TEM images of the droplets of oil-in-water emulsions were obtained using 10 µL of each diluted solution applied to a 400-mesh copper grid (carbon 400 mesh). The grid was kept under ambient conditions for 1 min, and the excess sample was eliminated using Whatman 41 filter paper. As a negative staining agent, we used 1% of staining uranyl acetate filtrated by 0.22 μm membrane (Merck-Millipore, USA), applied to the grid, and left to dry before obtention of the TEM images [28].

### Circular dichroism spectroscopy

Far-UV CD spectra of RBD and RBD-P were recorded at 37 °C with a JASCO J-720 spectropolarimeter (Jasco Inc, USA), as previously described [54]. Protein solutions containing ca. 0.1 mg/mL in pure water were loaded in a quartz cell with a path length of 1.0 mm. Spectra were recorded at 10 nm/min with a response time of 16 s. Each spectrum was corrected by buffer signal, corresponding to the average of three repetitive scans. Spectra are reported as mean residue ellipticity, [θ]_mrw_. Secondary structure content was estimated from CD spectra using the BeStSel deconvolution webserver [57].

### Recombinant RBD and RBD-P mass spectrometry analysis

The molecular mass of RBD and RBD-P was determined by MALDI-TOF, using a Bruker Microflex instrument equipped with a 20 Hz nitrogen laser at I = 337 nm. Approximately 500 fmol of solutions of RBD and RBD-P and α-cyano-4-hydroxycinnamic acid (10 mg/ml) (1:1 v/v) were mixed and applied on stainless steel plates. Samples were analyzed in linear and positive ion detection mode; 70 laser shots were integrated into a single mass spectrum.

### Western blot anti-RBD and anti-RBD-P

Proteins from the gel were transferred to a polyvinylidene difluoride (PVDF) membrane (Merck-Millipore, USA) using a semi-wet system (Bio-Rad, USA). The membrane was blocked with 5% (w/v) nonfat dry milk (Svelty, Nestle Mexico) and incubated with 1:2000 anti-S monoclonal antibody (Cat: 40591-MM43, Sino Biological, China). Three washes were carried out, followed by incubation with 1:5000 peroxide-conjugated affinipure goat anti-mouse IgG (H+L) (115-035-003, Jackson ImmunoResearch Labs, USA). Bands were detected by chemiluminescence with Super Signal West Pico chemiluminescent substrate (Thermo Fisher Scientific, USA) and visualized with the C-DIGIT blot scanner (LI-COR, USA). A homemade luminescent strip on top of the PVDF membrane was used to detect immunoreactive proteins placed in the same place as the SDS-PAGE molecular weight marker (ThermoFisher, USA).

### Recombinant RBD and RBD-P oil-in-water emulsion formulation

Water-solubilized proteins were incorporated into an oil-in-water emulsion for RBD and RBD-P oral dose administration to a final concentration of 0.1 mg/mL. The emulsion was formed by mixing edible safflower oil with saponin and tween 80 (Merk-Sigma-Aldrich, USA) using a sonicator. The emulsion was homogenized for 10 s on ice, during two homogenizer cycles (SoniPrepr150, Sanyo-Gallen-Kamp, United Kingdom) using an amplitude of 8 μm, with 30-s pauses on ice between each pulse.

### Animal, immunizations, and sample collection

To evaluate the in vivo activity of the proteins produced, a BALB/c mice model was used, employed in similar evaluations [58]. Mice were obtained from the Biological Models Unit at the Instituto de Investigaciones Biomédicas of the Universidad Nacional Autónoma de Mexico (IIB-UNAM). Animals were housed in groups of seven (males or females, independently) and were fed with standard diets. All animal studies were approved by the Institutional Committee for the Care and Use of Laboratory Animals (CICUAL at IIB-UNAM) in charge of ethical and responsible animal management (ID 7352).

Eight groups of seven mice were immunized orally, as previously described [47, 59–62]. BALB/c mice, females, and males were orally immunized with 100 µL of each oil-in-water RBD and RBD-P emulsion independently, using a soft adapted cannula that does not disturb the animal. The dose was administered two times, separated by fifteen days each, and compared with control mice (administered with 0.1 mL of PBS or the oil-in-water emulsion without proteins). Blood samples were collected before the first immunization and on day 45. Then, the animals were euthanized [63]. Furthermore, two intramuscular immunizations were performed at the same time as oral administration on six groups of BALB/c mice (n = 7). We used 0.1 mg/mL of each recombinant antigen purified in PBS, containing 10 μg/mouse of the adjuvant S6322-1VL (Monophosphoryl-lipid A + Trehalose dicorynomycolae, from Merck Sigma-Aldrich, USA), which is a stable oil-in-water emulsion widely used as a substitute to Freund′s adjuvants.

### Detection of recombinant RBD and RBD-P proteins

Enzyme-linked immunosorbent assays (ELISA) were used to detect polyclonal antibodies in sera from mice immunized with recombinant RBD and RBD-P. From each study group (females 7; males 7), 10 µL of serum was taken from each mouse. Each evaluation was performed by duplicate. The recombinant RBD was coated in flat bottom 96 well plates (Santa Cruz, USA) using 0.5 µg of protein in 100 µL of 1X PBS and incubated for 18 h at 4 °C. After washing by three times the plate with PBS + 0.05% Tween-20 (Merck, Germany) (PBST) for 5 min, a 2 h block was performed with PBS—0.05% tween—1% gelatin, followed by 2 h incubation with murine serum placed at a dilution 1:200 for intramuscular groups, and at a dilution 1:100 for oral groups, in 100 μL of PBS—0.1% gelatin. After being washed again in triplicate with PBST for 5 min, the secondary antibody peroxide-conjugated affinipure goat anti-mouse IgG (H+L) antibody (HRP) (115-035-003; Jackson ImmunoResearch Labs, USA) was used at 1:5000 for 1 h, and developed using 3,3ʹ,5,5ʹ-Tetramethylbenzidine (TMB; SigmaFast Cat: P9187-50Set, Merck-Sigma-Aldrich, USA) for 30 min. Nonspecific binding wells were incubated with 1xPBS and treated in the same way (negative control). The TMB reaction was stopped with 0.5 M H_2_SO_4_. The absorbance reading was performed at 450 nm (StatFax 4200, Awareness Technology Inc., USA).

Furthermore, an ELISA was performed to evaluate whether IgG antibodies from sera of convalescent SARS-CoV-2 patients recognized the RBD and RBD-P proteins. Also, twelve human sera obtained before the COVID-19 pandemic were used as control. The plate was coated with 0.5 µg of recombinant RBD or RBD-P protein. Subsequently, blocking was performed with PBST—2% gelatin, followed by the addition of human serum in a 1:100 dilution, and for detection, the secondary antibody HRP-conjugated goat anti-human IgG, Fc Fragment (A80-104P, Bethyl Laboratories. USA) at 1:100,000 dilution (done by sequential 1:100 and 1:1000, dilutions) was used. The reaction was developed using TMB. The TMB reaction was stopped with 0.5 M H_2_SO_4_. The plate was read at 450 nm (StatFax 4200, Awareness Technology Inc., USA). The control of each sample was PBS—blocking solution—human serum—secondary antibody—TMB. Twenty serum samples from no hospitalized individuals convalescing from coronavirus disease 2019 (COVID-19) (median age around 40 years) and after two months of SARS-CoV-2 infection were tested. Sample material was anonymized and integrated into a biobank using a protocol approved by the local ethics committee at the Biomedical Research Institute. Infection with SARS-CoV-2 was confirmed by RT-PCR from nasopharyngeal swabs performed in clinics.

### Sera recognition from mice immunized with RBD and RBD-P against recombinant RBD Wuhan and Omicron

The recognition of sera from animals immunized with oil-in-water emulsions containing RBD and RBD-P was tested against recombinant Wuhan (RBD-P) and Omicron (RBD-o) produced in *E. coli*. Briefly, 0.5 µg of recombinant RBD-P or RBD-o in 100 μL of PBS 1X was coated in an ELISA plate overnight at 4 ºC. Then, plates were washed three times with PBST (Merck, Darmstadt, Germany). Subsequently, blocking was performed with PBTS—1% Gelatin, followed by incubation with murine serum at a 1:100 dilution in 100 μL of PBS—0.1% gelatin. The secondary antibody used was peroxide-conjugated affinipure goat anti-mouse IgG (H+L) (115-035-003; Jackson ImmunoResearch Labs, USA) at a 1:5000 dilution and was revealed with TMB (ES001, Merck, USA). Control wells were treated in the same way but without RBD protein attached and only incubated with 1X PBS. The TMB reaction was stopped with 0.5 M H_2_SO_4_. The absorbance reading was performed at 450 nm (StatFax 4200, Awareness Technology Inc., USA).

### In vitro serum neutralization test

An ELISA was performed to identify the neutralizing antibodies in murine sera immunized orally with oil-in-water emulsions containing recombinant RBD and RBD-P. Ten microliters of mouse serum diluted with sample dilution buffer were incubated for 30 min at 37 °C with HRP-conjugated RBD protein produced in human cells, as indicated by the supplier (Catalog Z03594, Genscript, Ryswick, The Netherlands). 100 µL of the sample were placed on a capture plate with hACE2 protein from the cPass SARS-CoV-2 Surrogate virus neutralization test kit (Catalog L00847-A, Genscript, Ryswick, The Netherlands) for 15 min at 37 °C. Four washes were performed, and the reaction was revealed with 100 µL of TMB, incubated for 15 min in the dark. The reaction was stopped and read at 450 nm (StatFax 4200, Awareness Technology Inc., USA) [64, 65].

### IgG titer determination

ELISAs were performed to assess the production of antibodies. Ultra-Cruz ELISA Plates (Santa Cruz Biotechnology, USA) were coated with 0.5 μg of RBD recombinant protein from *E. coli*, in 100 μL of PBS 1X. The coating solution was incubated overnight at 4 °C. The plate was washed three times with PBST (Merck, Germany). Next, 200 μL of PBS 1X—1% gelatin from cold water fish skin (Merck-Sigma-Aldrich, USA) was used for blocking and incubated for 1 h. The plate was washed three times with PBST by 5 min. Using serum from mice, the following dilutions were made: 1:50, 1:100, 1:200, 1:400, 1:800, and in 100 μL of PBS 1X—0.1% gelatin and incubated for 2 h. Each dilution was placed in duplicate on the plate. The plate was washed three times with PBST by 5 min. Then, 100 μL of peroxide-conjugated affinipure goat anti-mouse IgG (H+L) (115-035-003; Jackson ImmunoResearch Labs, USA) at a dilution of 1:5000 was added and incubated for 1 h. The plate was washed three times with PBST. TMB substrate (Merck-Sigma-Aldrich, USA) was used and incubated for 30 min in the dark to reveal the ELISA reaction. The TMB reaction was stopped with 0.5 M H_2_SO_4_. The absorbance was measured on a microplate reader (Stat Fax® 4200, Awareness Technology, Inc. Palm City, FL, USA) at 450 nm. The antibody titer was calculated using the EC50 of the absorbance obtained with different dilutions used.

### Statistical analyses

All the data for kinetic parameters are represented as the mean of triplicates ± standard deviation. One-way ANOVA for independent and samples and, when needed, pair-wise comparisons using Tukey HSD (Test for Post-ANOVA) were carried out.

Statistical significance between groups in the antibody reactivity assay was determined using sera from mice and compared with PBS as the control. Dunn´s test was used for all pairwise comparisons and comparisons against a control group (PBS) following rank-based ANOVA based on the treatment of unequal group sizes. Differences were considered statistically significant if the p < 0.05.

## Results

### Production of RBD and RBD-P in shake flasks and bioreactor

**Fig. 1:**
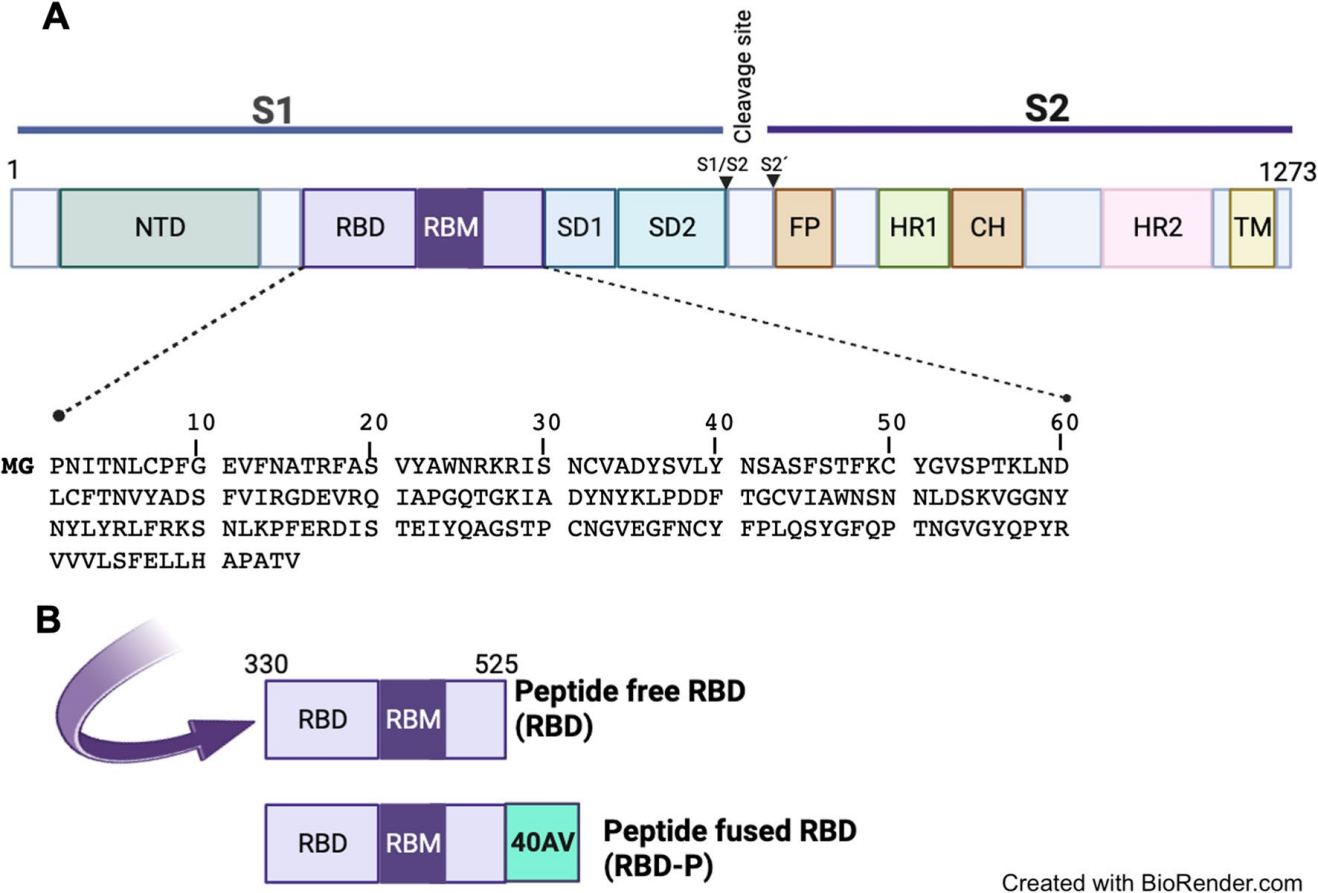
Scheme of Spike (S) protein architecture of the SARS-CoV-2 (**A**): NTD N-terminal domain, RBD receptor-binding domain (330–525 WA1/2020), RBD-P receptor-binding domain fused to 40AV peptide, RBM receptor binding motif, SD1 subdomain 1, SD2 subdomain 2, S1/S2 protease cleavage site, S2' protease cleavage site, FP fusion peptide, HR1 heptad repeat 1, CH central helix, HR2 heptad repeat 2, TM transmembrane domain. Representative scheme of the peptide fused RBD and peptide free RBD (**B**)

We design a peptide for fusion with the RBD domain of SARS-CoV-2, aiming to enhance antigen presentation that can be orally administered in an oil-in-water emulsion. The gene sequence encoding the RBD (597 bp) region contained in the S gene of SARS-CoV-2 (GenBank: MN908947) was synthesized using the preferential codon usage in *E. coli* [66]. Codons for the amino acids MGKL were added at the 5ʹ of the sequence, whereas codons for the peptide of 40 amino acids were added at the 3ʹ of the sequence (Fig. 1). The peptide named 40AV was fused with the RBD region and compared with the RBD without fusion peptide. Both plasmids coding for each construction were cloned in the plasmid pET15b and used to transform *E. coli* BL21 (DE3) (Merck-Novagen, USA). Constructions with and without the peptide were named as RBD-P and RBD, respectively. The constructed plasmids were verified by PCR and sequencing. Ampicillin was used to select recombinant bacteria, and master and working cell banks were produced from each strain.

**Fig. 2:**
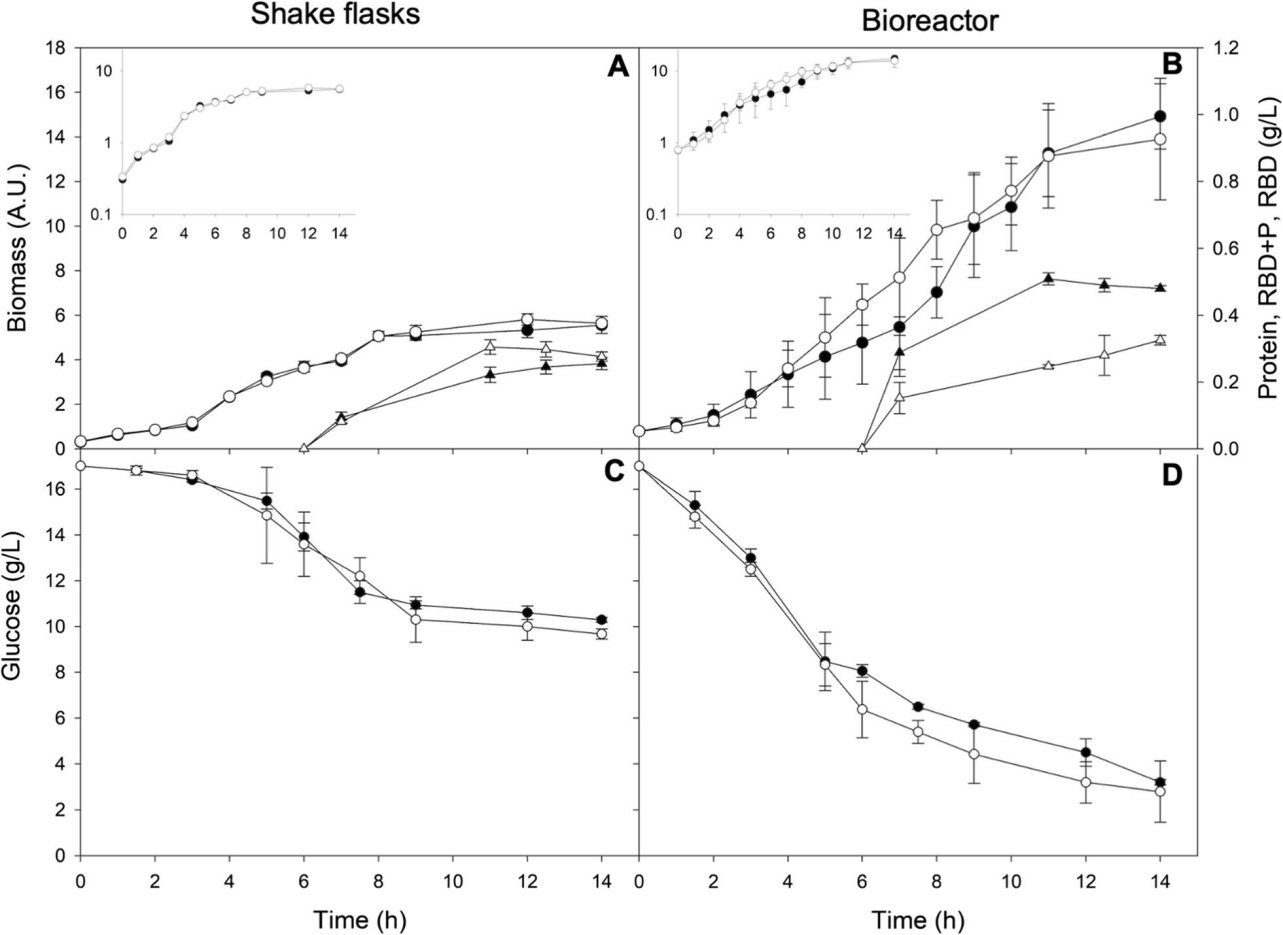
Kinetics of biomass growth (circles) and recombinant protein production (triangles) of RBD-P (filled symbols) and RBD (open symbols) from SARS-CoV-2 by *E. coli* BL21 (DE3), in shake flasks (**A**) and in bioreactors (**B**). Data presents the average and standard deviation of the cultures carried out at least in triplicate. Induction with IPTG started after 6 h of culture. Inset figures are logarithmic biomass growth. Glucose consumption was also presented for shake flasks (**C**) and bioreactors (**D**) cultures

Selected clones were grown in Erlenmeyer flasks in triplicate. The cultures producers of RBD-P and RBD reached similar maximum biomass (5.56 ± 0.39 A.U. and 5.81 ± 0.25 A.U., respectively). However, there were significant differences in the specific growth rate (µ) of *E. coli* BL21 (DE3) producing RBD-P (0.49 ± 0.01 h^−1^) and RBD (0.45 ± 0.01 h^−1^) (Fig. 2A, Table 1).

**Table 1:** Stoichiometric and kinetic growth parameters of *E. coli* BL21 (DE3) producer of RBD-P and RBD growing in shake flasks and bioreactor

Parameter	Shake flasks		Bioreactor	
	RBD	RBD + P	RBD	RBD + P
^A^µ (h^−1^)	0.45 ± 0.01^a^	0.49 ± 0.01^b^	0.41 ± 0.03^c^	0.42 ± 0.01^c^
^B^X_max_ (A.U.)	5.81 ± 0.25^a^	5.56 ± 0.39^a^	13.90 ± 2.73^b^	14.91 ± 1.46^b^
^C^Glucose consumed (g/L)	7.3 ± 0.2^a^	6.7 ± 0.1^b^	14.2 ± 1.7^c^	13.8 ± 0.2^d^
^D^Y_X/S_ (g/g)	0.21 ± 0.02^a^	0.20 ± 0.01^a^	0.26 ± 0.01^b^	0.24 ± 0.02^b^
Soluble protein (g/L)	0.066 ± 0.028^a^	0.087 ± 0.027^a^	0.113 ± 0.014^b^	0.169 ± 0.034^c^
Insoluble protein (g/L)	1.088 ± 0.112^a^	1.039 ± 0.092^a^	0.871 ± 0.288^b^	1.651 ± 0.371^c^
Total protein (g/L)	1.154 ± 0.132^a^	1.126 ± 0.116^a^	0.930 ± 0.323^b^	1.819 ± 0.413^c^
^E^RBD (g/L)	0.28 ± 0.01^a^	0.26 ± 0.02^a^	0.33 ± 0.01^b^	0.46 ± 0.01^c^
^F^RBD in total protein (%)	24 ± 4^a^	23 ± 5^a^	35 ± 5^b^	25 ± 4^a^
^D^Y_P/X_ (g/g)	0.14 ± 0.02^a^	0.13 ± 0.02^a^	0.07 ± 0.02^b^	0.09 ± 0.01^b^

The values represent the mean and standard deviation for three biological replicates per condition. The statistical differences are indicated with different letters (p < 0.05)

A: The specific growth rate was calculated from the slope of growth in the exponential growth phase

B: The maximum biomass was obtained where the biomass was the maxima, regardless of the culture time

C: Glucose consumed is the initial glucose calculation subtracted from the residual glucose from Fig. 2C and D

D: Y_X/S_ and Y_P/X_ were calculated from glucose consumption until the X_max_ in each condition

E, F: Obtained from densitometric analysis of SDS-PAGE gels of at least three biological replicates per condition

*E. coli* BL21 (DE3) clones producing RBD-P and the RBD were also characterized in bioreactor cultures (Fig. 2B). The µ of *E. coli* BL21 (DE3) cultures, either producing the RBD-P or RBD, was 0.42 ± 0.01 h^−1^, whereas maximum biomass of 14.91 ± 0.10 A.U. and 13.9 ± 2.73 A.U. was reached for cultures producing the RBD-P or RBD, respectively (Fig. 2B, Table 1). At the end of the cultures, the maximal biomass reached in the bioreactors was more than twice in both cultures, producing RBD-P and RBD, compared to those in shake flasks (Table 1). In shake flasks, only about 40% of the glucose was consumed in all cultures (Fig. 2C, Table 1), while in bioreactors, the carbon source was almost completely consumed (Fig. 2B, Table 1). No significant differences were observed in the biomass per glucose yields (Y_X/S_) between cultures of RBD-P and RBD. Instead of, Y_X/S_ was higher in bioreactor cultures than in shake flasks (Table 1).

### RBD and RBD-P identification, quantification, and secondary structure analysis

**Fig. 3:**
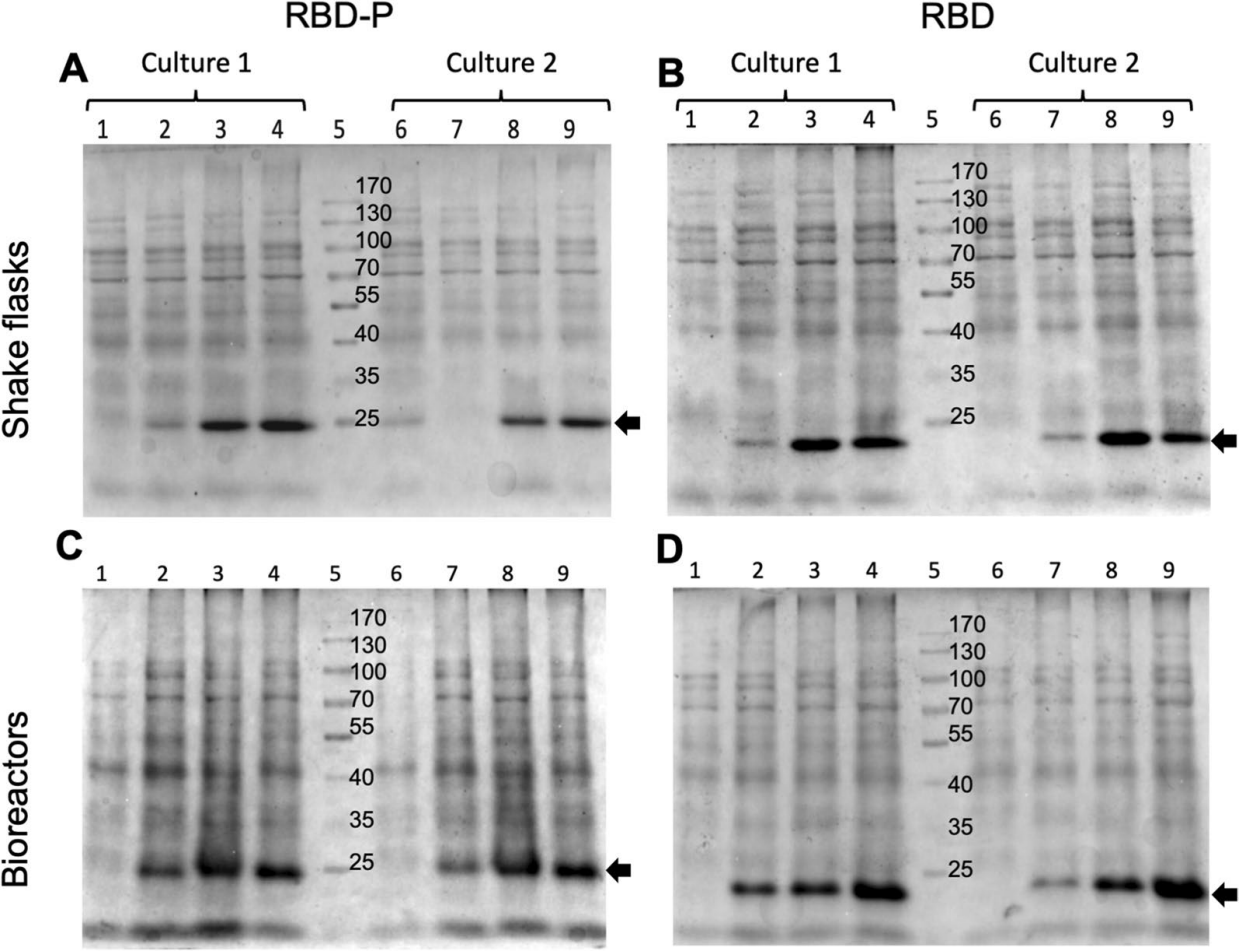
Kinetic comparison in SDS-PAGE (12%) of total protein (TP) produced in cultures of *E. coli* BL21 (DE3) grow up in shake flasks (**A**, **B**) and bioreactors (**C**, **D**). RBD-P producing cultures are shown in (**A)** and **(C**) and RBD in (**B**) and (**D**). In all gels, lanes 1 and 6 correspond to PT before induction. Lanes 2, 3, and 4 (7,8, and 9) show samples taken from cultures at 1, 5, and 10 h after induction, respectively. Lane 5 corresponds to the molecular weight marker (MWM). Lanes 6 to 9 are samples from replica experiments. Recombinant proteins are marked by a black arrow

Final total protein (TP) content, after IEF solubilization, was 1.154 ± 0.132 g/L and 1.126 ± 0.116 g/L in shake flasks producers of RBD and RBD-P, respectively, and 0.930 ± 0.323 g/L and 1.819 ± 0.413 g/L, in bioreactors cultures producing RBD or RBD-P, respectively (Table 1). RBD and RBD-P concentrations were determined by densitometry in total protein fractions analyzed on 15% SDS-PAGE. Samples at 1 h, 5 h, and 10 h post-induction times collected from shake flasks and bioreactors were loaded in gels and compared with a sample harvested before induction and used as a negative control (Fig. 3). In agreement with the theoretical molecular weights, RBD and RBD-P were observed at around 22 and 26 KDa, respectively (Fig. 3), and confirmed by intact mass analysis (22,738.12 and 26,490.03 u.m.a., respectively), with around 3 to 5 Da higher than the theoretical mass (Additional file 1: Figure S1A).

**Fig. 4:**
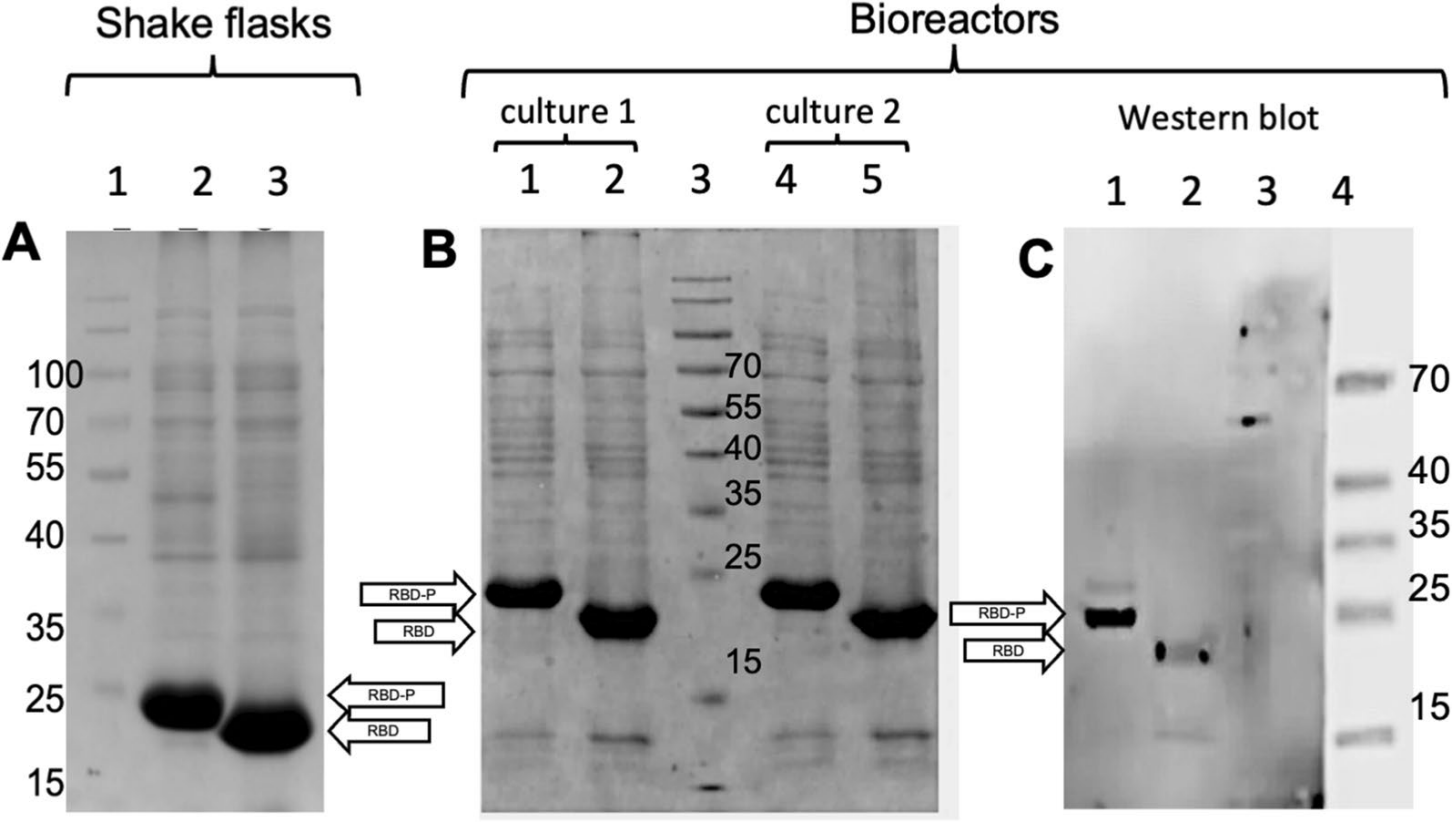
Coomassie blue stained 12% acrylamide gel electrophoresis of the IBs obtained at the end of *E. coli* BL21 (DE3) cultures producers of RBD-P and RBD. IBs from cultures in shake flasks (**A**); lane 1: molecular weight marker (MWM), lane 2: IBs of RBD-P, and lane 3: IBs of RBD. IBs from two independent bioreactor cultures (**B**); lanes 1 and 4 are IBs from cultures producers of RBD-P, lane 3 is the MWM, and lanes 2 and 5 are IBs from cultures producers of RBD. Immunodetection by Western Blot of purified RBD-P and RBD (**C**) recombinantly produced in *E. coli* BL21 (DE3) in bioreactor and detected with anti-Spike antibody (Sino Biological 40,591-MM43); lane 1: RBD-P, and lane 2: RBD, and lanes 3 and 4: molecular weight marker (MWM)

As has been described for many bioprocesses, a higher accumulation of recombinant proteins was observed in bioreactors (RBD: 330 ± 10 mg/L and RBD-P: 460 ± 10 mg/L) compared to shake flasks (RBD: 280 ± 10 mg/L and RBD-P 260 ± 20 mg/L) (Table 1). Almost all the recombinant proteins were accumulated in the insoluble fraction, particularly forming protein aggregates or inclusion bodies (IBs). After cell rupture, IBs were isolated and solubilized, and proteins were separated by gel electrophoresis. RBD and RBD-P from a shake flask and bioreactor showed a high accumulation of proteins with 22 and 26 KDa, respectively (Fig. 4A and B). The identity of RBD and RBD-P was verified by immunodetection in Western blots using a specific anti-Spike antibody (Fig. 4C). The content of recombinant proteins in IBs, estimated by densitometry, corresponded to around 24% for both (RBD and RBD-P) accumulated in shake flasks, while in bioreactors, it corresponded to around 35% for RBD and 25% for RBD-P (Table 1). In addition, the composition of IBs containing RBD or RBD-P was different, as inferred by the band patterns (Fig. 4). Intracellular IBs, produced after 5 h induction with IPTG, measured up to 800 nm and had pseudo-spheric and irregular shapes (Additional file 1: Figure S2). At least 40% of the cell population contained IBs, and around 25% of cells contained two or more IBs.

**Fig. 5:**
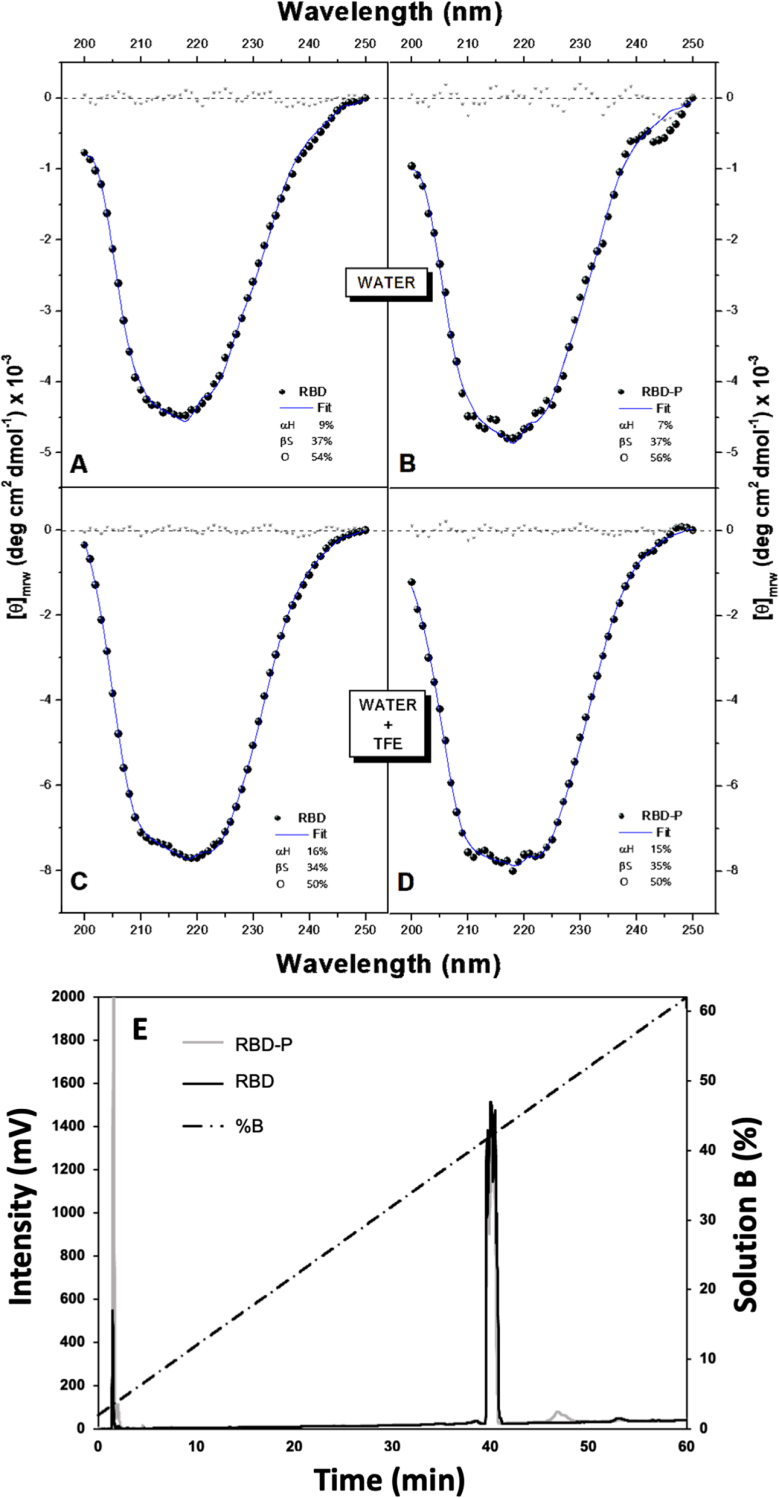
Secondary structure characterization of RBD forms by CD spectroscopy. The spectra were recorded in pure water **A** RBD and **B** RBD-P and in a 50% (v/v) TFE aqueous mixture, **C** RBD and **D** RBD-P. Secondary structure contents (αH, α helix; βS, β strand; O, other) were calculated from a deconvolution analysis of the CD spectra with the BeStSel webserver. Black circles are experimental spectra, lines correspond to the best-fit spectra, and residuals between calculated and experimental data are shown with asterisks. High-resolution chromatography of the RBD-P and RBD antigens on an Xbridge Protein BEH C4 reverse phase column (**E**). The interest components were eluted at 39.6 min, in a gradient from 0 to 60% acetonitrile in 60 min, with a flow of 0.8 mL/min, and injected 25 μg

The recombinant proteins produced in the bioreactor and isolated from IBs were solubilized, separated by gel chromatography, and recovered in MQ water by electro-elution [54]. Around 50% of the recombinant proteins were recovered. Then, 25 µg of RBD and RBD-P were separated by high-resolution chromatography. The resultant components had a purity of 90 and 85% for RBD and RBD-P, respectively, and were eluted at 39.6 min, in a 0 to 60% gradient of acetonitrile in 60 min, with a flow rate of 0.8 mL/min (Fig. 5A).

RBD and RBD-P were characterized by CD spectroscopy to determine their conformational properties. RBD and RBD-P showed basically the same spectrum, indicating that the fusion peptide does not affect RBD conformation, at least in terms of secondary structure (Fig. 5). The shapes of these spectra mirrored that reported for RBD also produced in *E. coli*, although those obtained with eukaryotic cells are markedly different [67]. A deconvolution analysis indicated that the spectra are consistent with the presence of 7–9% α helix and 37% β strands (Fig. 5B and C). Since the optical properties of the oil-in-water emulsion did not allow the spectra to be determined, RBD and RBD-P were solubilized in 50% TFE (Tetrafluoroethylene, Merck-Sigma-Aldrich, USA) / water (v/v) solution, a cosolute used to mimic the environment composed of lipids. In the presence of TFE, the two proteins also exhibited similar spectra to each other. In agreement with what was observed in pure water, these results again indicated that the peptide does not interfere with the conformation adopted by RBD. Under these conditions, the two RBD forms presented a moderate enrichment of α helix (15–16%) without significant detriment of the β-strand content (34–35%). The composition in TFE/water was closer to that observed in the native structure of RBD (20% α helix and 23% β strand), although still different from the biologically active conformation [27].

### Immunization of RBD-P and RBD via oral and intramuscular

**Fig. 6:**
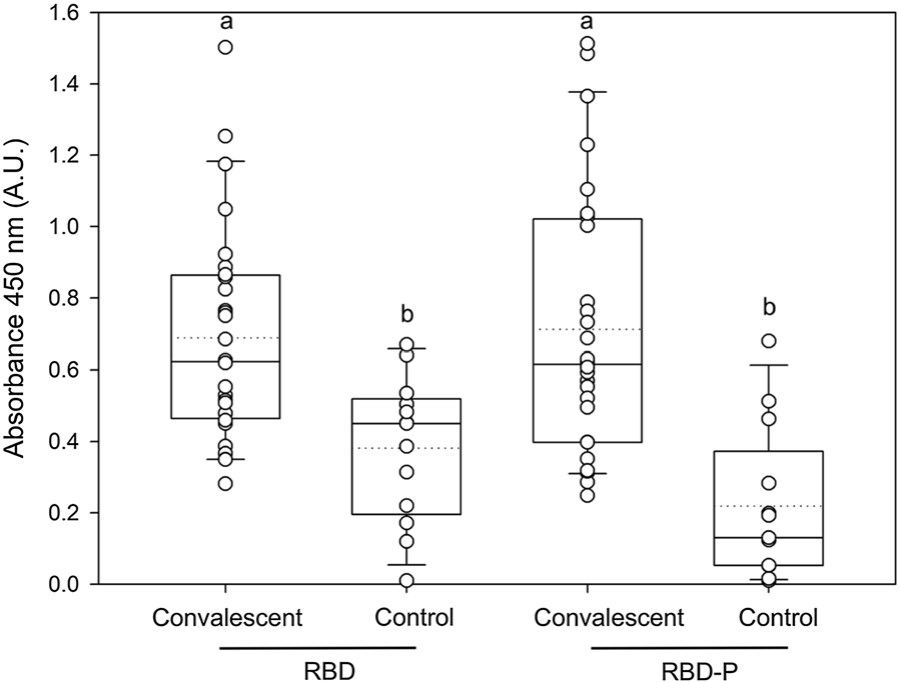
Recognition of recombinant RBD and RBD-P by human IgG polyclonal antibodies. The ELISA was performed using sera samples from no hospitalized individuals convalescing from COVID-19 (median age around 40 years). Also, twelve human sera obtained before the COVID-19 pandemic were used as control. Infection with SARS-CoV-2 was confirmed by RT-PCR from nasopharyngeal swabs performed in clinics. In boxplots, each data point is the mean and its deviation of replica per serum. Also, each boxplot presents the mean in a dotted line, the median as the middle line, the interquartile range as box limits, and the 2.5th and 97.5th percentiles as the whiskers. For all variables with the same letter, the difference between the means is not statistically significant. If two variables have different letters, they are significantly different (p < 0.05)

To analyze the nature of the recombinant RBD and RBD-P antigens, recognition by IgG from sera from COVID-19 patients, which present anti-SARS-CoV-2 antibodies, was evaluated, a comparison was made with human sera obtained before the COVID-19 pandemic. 71% and 78% of sera showed a signal greater than 0.5 A.U. in recognition of RBP and RBD-P, respectively (Fig. 6). No significant differences in recognition were found between the recombinant RBD-P and RBD (Fig. 6), but with controls. No neutralization was found from human sera obtained before the COVID-19 pandemic (data not shown).

**Fig. 7:**
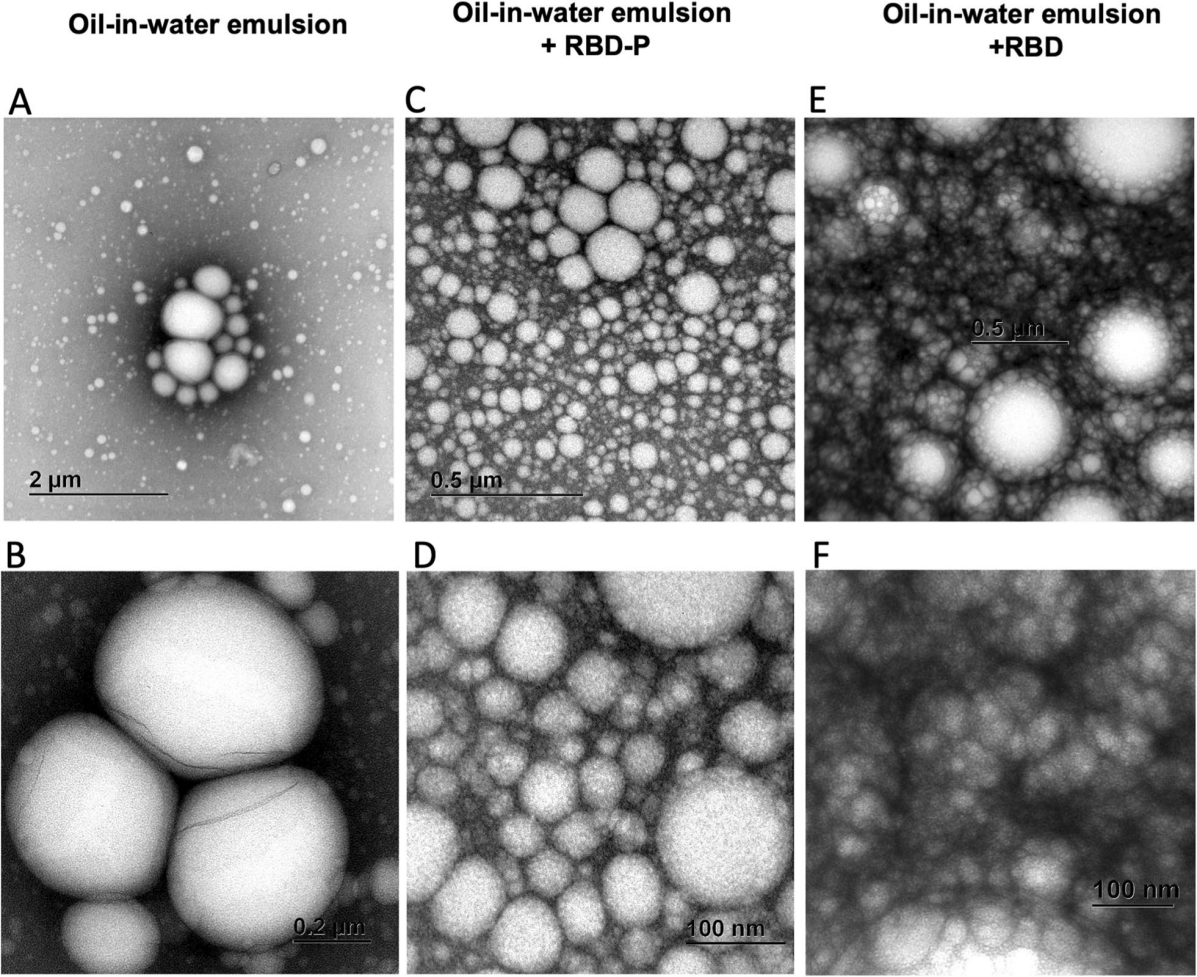
Micrographs by TEM of the oil-in-water emulsion without recombinant proteins (**A**, **B**), oil-in-water emulsion with RBD-P (**C**, **D**) and oil-in-water emulsion with recombinant RBD (**E**, **F**) developed for oral administration. Scale bars are found inside the micrographs

The recombinant antigens purified from bioreactor cultures were mixed with an oil-in-water emulsion to be administered orally aimed to increase the antigenicity. The developed oil-in-water emulsion presents droplets between 20 nm and 0.6 µm observed by TEM (Fig. 7). Interestingly, the emulsion containing 10 µg of RBD or RBD-P formed a series of nanodrops that decorated larger droplets, while nanodrops were absent around the droplets formed at the control oil-in-water emulsion without recombinant proteins. Furthermore, the oil-in-water emulsion containing RBD-P presented fewer nanodrops around larger droplets than RBD (Fig. 7).

**Fig. 8:**
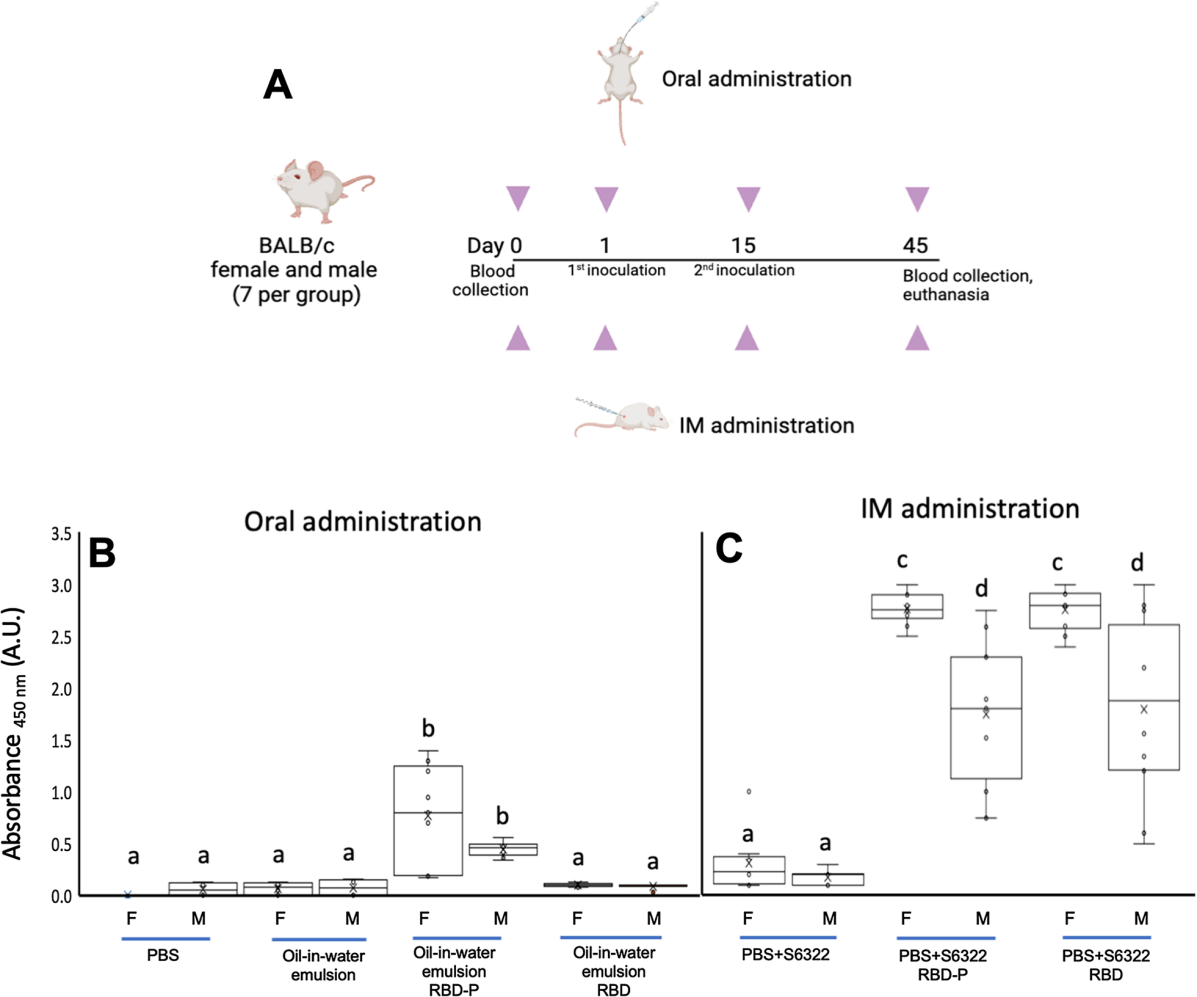
Immunization scheme of BALB/c mice with RBD-P and RBD (**A**). Analysis of antigen recognition with polyclonal antibodies of murine serum immunized with recombinant RBD and RBD-P orally (**B**) and intramuscularly (**C**). The Y axis shows the absorbance value measured at 450 nm Serum was placed at a dilution 1:200 for intramuscularly groups, and at a dilution 1:100 for orally groups. In boxplots, each data point is the mean of two wells per mouse serum. Also, each boxplot presents the mean as an X, the median as the middle line, the interquartile range as box limits, and the 2.5th and 97.5th percentiles as the whiskers. For all variables with the same letter, the difference between the means is not statistically significant. If two variables have different letters, they are significantly different (p < 0.05). The immunization scheme was prepared using Biorender

Oral doses were prepared to determine if this preparation could improve the antigenicity. The oral administration was compared with intramuscular doses (Fig. 8). RBD and RBD-P antigens were inoculated in BALB/c mice. A total of 14 groups of mice (seven animals per group) were evaluated (Fig. 8). All groups received two immunizations administered 15 days apart. We immunized with 0.4 µg/g of mice (10 µg per mice of 25 g) with purified antigens (RBD and RBD-P) in oil-in-water emulsion. Mice sera were obtained 30 days after the immunization period and used to evaluate RBD recognition by ELISA (Fig. 8B and C). The orally inoculated mice with RBD presented similar values to the control groups (oil-in-water emulsion without recombinant proteins or PBS), but RBD-P showed a significantly higher response (p < 0.001). No significant differences were found when comparing RBD-P immunization and antigen recognition by gender (Fig. 8B). Significantly, the oral administration of RBD did not promote IgG recognition against RBD, with similar results between males, females, and negative controls (Fig. 8B). In the intramuscular application, significant differences (p < 0.001) were found between females and males in both treatments (RBD-P and RBD), showing both treatments greater recognition compared to controls (Fig. 8C). Also, reactivity levels were like those reported in intramuscular administration [30]. Significant differences in the absorbance values between oral and intramuscular applications were found when comparing the RBD-P and RBD treatments (Fig. 8B and C). Therefore, the fusion peptide developed increases protein immunogenicity, probably due to an enhanced antigen presentation of the RBD domain in oral administration (Fig. 8B).

**Fig. 9:**
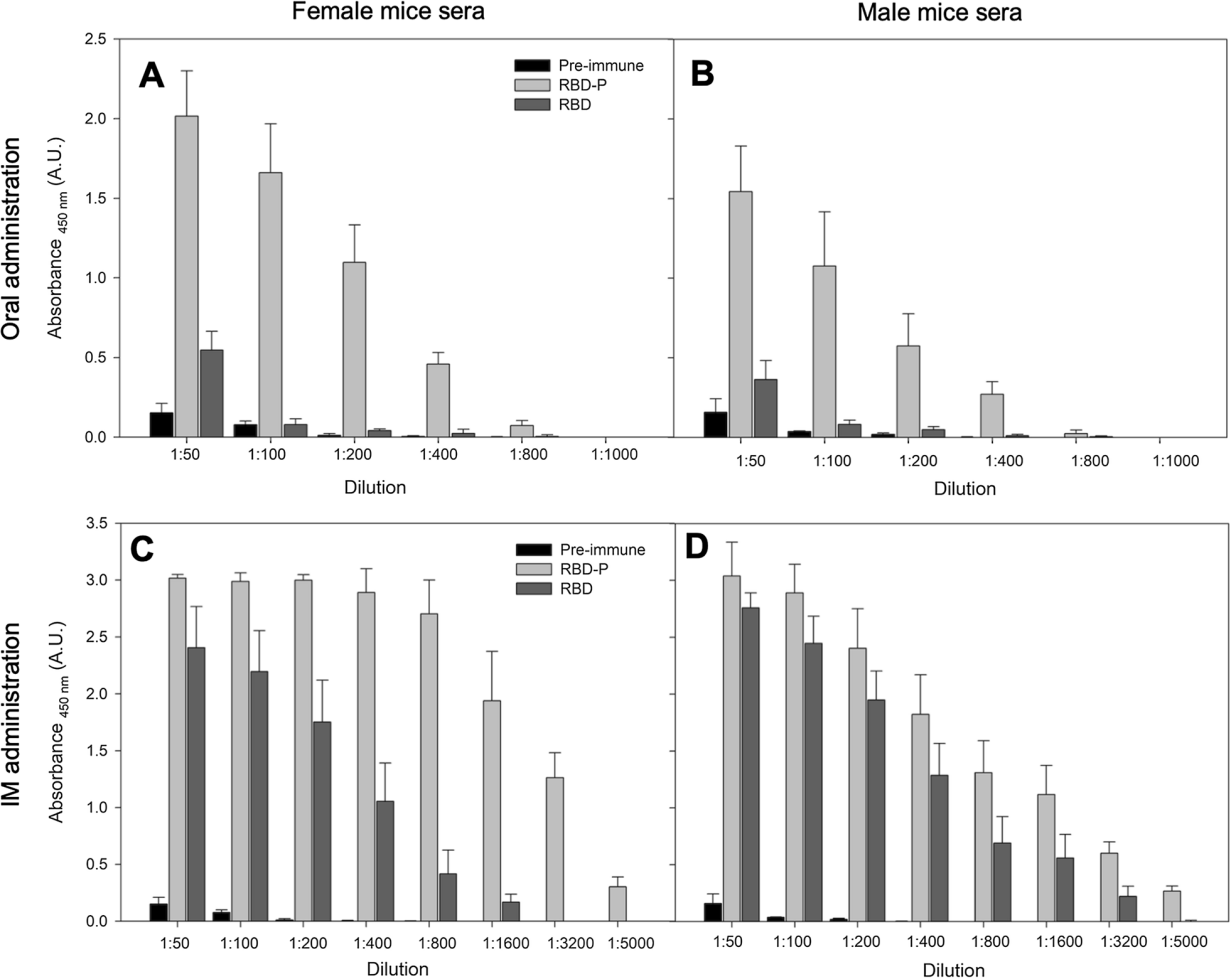
Indirect ELISA titration against recombinant RBP from *E. coli* of pre-immune (black) and hyperimmune female (left) and male (right) mice sera using RBD (grey) and RBD-P (light grey) antigens administrated orally (**A**, **B**) and intramuscularly (**C**, **D**). Results are presented as the mean and standard deviation of at least triplicates. There is a significant difference (p < 0.05) in all cases when the results using RBD (grey) and RBD-P (light grey) are compared for each dilution evaluated

The IgG quantification in immune murine plasma showed recognition of the RBD recombinant antigen at different dilutions (Fig. 9). Higher titration was obtained using serum from mice inoculated intramuscularly using RBD-P and RBD compared with those orally administrated. Importantly, with oral an IM administration, significantly higher titration (p < 0.05) was obtained with RBD-P compared with RBD (Fig. 9). The EC50 in the different titrations for oral administration was 1:200 for females and males, whereas, for intramuscular administration, it was 1:3200 and 1:800 for females and males, respectively.

Additionally, it was observed that both RBD-P and RBD-o were recognized by the IgGs present in the sera of immunized mice with RBD-P or RBD. A similar recognition pattern was observed when using the same amounts of sera and the same amount of bound protein in the ELISA test (Additional file 1: Figure S3). In the female sera, we observed a significant increase (p < 0.05) in recognition compared to the absorbance values obtained using the male sera (Additional file 1: Figure S3).

### RBD-P immunization in mice elicited neutralizing activity

**Fig. 10:**
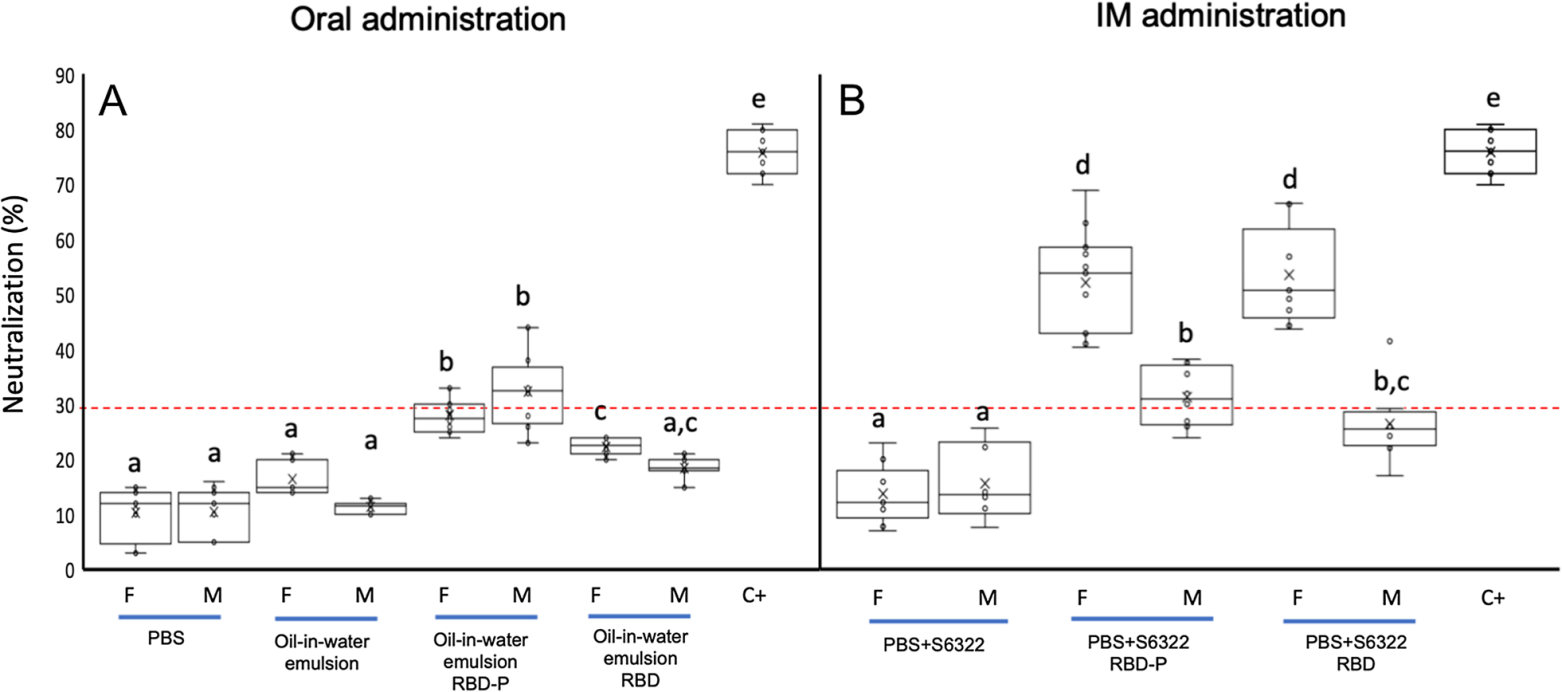
Neutralization assay of the hyperimmune sera obtained 30 days post-immunization of BALB/c mice with RBD-P and RBD orally (**A**) and intramuscularly (**B**). The y-axis corresponds to the observed percentage of the inhibition of HRP-conjugated RBD interaction with hACE2. Dashed lines represent manufacturers’ cutoff values (30%). The neutralization assay was performed in duplicate for each mouse serum, and per group. C+ is the positive control. Each boxplot presents the mean as an X, the median as the middle line, the interquartile range as box limits, and the 2.5th and 97.5th percentiles as the whiskers. For all variables with the same letter, the difference between the means is not statistically significant. If two variables have different letters, they are significantly different (p < 0.05). When there are two letters in a data set, this is not significantly different from the data sets with those letters

The hyperimmune sera obtained after 30 days post-inoculation were tested. The blockade percentage of the interaction between the commercial RBD and the hACE2 receptor was determined in a commercial neutralization test using murine polyclonal antibodies from each serum, obtained from oral and intramuscular immunization with recombinant RBD-P and RBD (Fig. 10). The percentage of inhibition is shown on the y-axis, and at least six biological replicates were plotted. The positive control included an anti-spike antibody, and this test was factory-calibrated (GeneScript, USA). It was observed that sera of RBD-P orally immunized mice produced antibodies that exhibited inhibition. Data showed no significant differences between evaluated females and males (Fig. 10A). Furthermore, neutralization for RBD-P ranged from 25 to 36% in orally immunized mice against 3% to 20% in the control groups (PBS and the oil-in-water emulsion). At the same time, RBD immunization presented below neutralization values than RBD-P (Fig. 10A).

The blockage percentage of murine polyclonal antibodies obtained from intramuscular immunization with RBD-P and RBD was statistically similar by gender. Moreover, in the intramuscular application, significant differences (p < 0.01) were found between females and males in both treatments (RBD-P and RBD), being greater for females and with neutralization averages greater than 50%, with higher values when compared with oral applications (Fig. 10A and B).

## Discussion

Until now, the vaccines available to treat COVID-19 are administered by injection, and the antibodies have not been long-lasting. Previous studies have shown a reduction in vaccine effectiveness over time [11, 68], so it has been proposed to continue with periodic vaccination according to the variants that cause waves of population infections [68]. More than 300 molecules are under study for the development of new vaccines around the world. The approaches to improve the presentation of antigens become relevant when vaccines, such as those against viruses like SARS-CoV-2, will have to be used periodically for a long time.

The RBD region is one of the most important domains of interaction with the virus receptor hACE2 and its antigenicity [25, 27]. This domain has qualities of movement between open and closed states and has been suffering mutations that cause new variants with different affinities for the receptor [11]. This work has developed a technology with possible utility in the treatment of COVID-19. In the present study, we produced recombinantly in *E. coli* the RBD and the RBD-P, the last containing an extension consisting of one peptide of 40 amino acids to enhance RBD immunogenicity by integrating a product of the fusion of two parts. One part exhibits inherent flexibility, while the other draws inspiration from peptides forming amphipathic helices [69], strategically tailored to foster interaction with cell membranes. This innovative design aimed to incorporate the RBD-P in oil-in-water emulsion for oral administration, with the goal of eliciting the production of neutralizing antibodies. Future studies are necessary to determine the action mode of the 40AV peptide.

The RBD and RBD-P production was performed in shake flask and bioreactor cultures where, in the last, the recombinant protein accumulation was higher, and oxygen limitation was avoided [70–74]. Whatever, all cultures accumulated more than 250 mg/L of RBD and RBD-P, a higher accumulation compared with other works producing RBD-like proteins [75, 76]. Recombinant antigens produced in shake flasks and bioreactors formed aggregates (inclusion bodies) (Additional file 1: Figure S2) that were solubilized and purified. The prokaryotic production of the RBD of SARS-CoV-2 has presented the problem of protein aggregation and IBs formation due to the high number of cysteines, the inefficient formation of disulfide bonds, and high recombinant productivity [75, 76]. Therefore, solubilization of IBs and refolding is common and has allowed the obtention of biologically active RBD proteins [30, 77]. Here, we confirmed the antigenic determinants of RBD and RBD-P by immunoassays using a specific antibody anti-Spike and by recognition by sera from human patients infected by SARS-CoV-2. Differential recognition between sera from infected and pre-pandemic people was found, denoting that both antigens present elements and domains like those in the SARS-CoV-2. In addition, both antigens were characterized by SDS-PAGE and mass spectrometry, confirming their molecular weight. CD spectroscopy analysis revealed that the 40AV peptide extension had an insignificant impact on the secondary structure content of the RBD, both in an aqueous medium and in a water/TFE mixture designed to emulate membrane environments. This opens the possibility that the role it plays in the observed increase in antigenicity is to allow RBD to be recruited in lipid environments such as the oil-in-water emulsion here developed and in vivo, probably facilitating its presentation to the immune system when RBD-P was administered orally.

The antigens produced in bioreactors under controlled conditions were administered in mice. Only the groups inoculated with RBD-P orally showed antigenicity and immunogenicity. The edible formulation, the oil-in-water emulsion containing RBD or RBD-P, showed no toxic effects, no reactive episodes, and no changes in temperature or vomiting in mice. Moreover, after each administration, the animals ate food normally. Immunization with 10 μg of RBD-P per BALB/c mice, formulated in the oil-in-water emulsion, induced a robust IgGs response capable of recognizing recombinant RBD proteins through ELISA. Furthermore, since the RBD is the outermost region of the virus and mutations can define its interaction with the hACE2 receptor [22, 23], our results showed that RBD and RBD-P stimulate the production of IgG in the serum of experimental animals, in which IgGs produced recognize both the Wuhan and Omicron BA.1 RBDs (Additional file 1: Figure S3). Aparicio et al., [78] found antibodies induced by Wuhan RBD peptide in mice cross-react with Omicron peptides. Less recognition was observed in sera obtained from males compared to females, although the recognition profile, in general, is like the recognition of the Wuhan RBD and Omicron (Additional file 1: Figure S3). Interestingly, the differences observed in the production of recognition antibodies in terms of gender have been scarcely discussed. In humans, it has been reported that females have better antiviral immune mechanisms [79–82], while in BALB/c mice models, males have been shown to be more susceptible than females to SARS-CoV infection because estrogens and estradiol seem to enhance immunity to viral infections [83]. In agreement with the findings of this study, other authors have described a differential immune response to viral vaccines between male and female BALB/c mice, with higher responses in females [84, 85]. For example, there are higher CD4 T cell counts in females immunized with immunodominant glycoprotein of Herpes simplex type 1 [84]. Furthermore, the trivalent influenza vaccine induces greater titers of IgM and IgG in female mice than in males [85]. The above is explained by sex-related immune characteristics. Polymorphism of immune-associated genes located in X chromosome results in more cell diversity in females than males [86, 87], with differences in TLR7/8 and type 1 interferon production, crucial in viral infections [88], cytokine modulation [84] and MicroRNAs, involved in granulocyte generation and maturation [89, 90], among other immune activities [91].

Outstandingly, serum from mice inoculated with RBD-P produced antibodies after two oral immunizations that blocked the RBD binding to the hACE2 receptor in an in vitro test, causing the generation of neutralizing antibodies. It is important to mention that the difference observed in the magnitude of the IgG isotype between oral and intramuscular administration was expected since the intramuscular route of administration induces a strong serum IgG response [30, 92, 93]. The IgA immunoglobulins are known to facilitate the humoral response in mucosal tissues, but it has also been demonstrated that IgM and IgG contribute to mucosal-associated immunity [41, 47]. Although oral administration did not induce high titers of neutralizing IgG antibodies in serum and measurement of IgA is still pending in the present work, mucosal administration of SARS CoV-2 immunogens is a promising research target, considering that mucosal immunity promotes effective disease prevention and avoids viral transmission [94, 95]. Due to the peptide-coupled polymeric nature of the present vaccine prototype, a good mucosal response could be expected. However, if oral immune responses needed to be enhanced, nanoparticles [96], mucosal adjuvants such as *E coli* double mutant thermolabile toxin (dmLT) [97, 98], multiple mutated choleratoxin (mmCT) [99] α-galactosylceramide (α-GalCer) [100], chitosan [101], among others could be used [102–104]. The administration of oral vaccines involves the use of substantial amounts of antigen to counteract its degradation in the gastrointestinal tract [103]. Our results suggest that the oil-in-water emulsion formulation prevents degradation. Furthermore, tolerability and response experiments on the mice can be carried out at higher doses of the antigens described here.

The unglycosylated RBD from SARS-CoV and SARS-CoV-2 could elicit the cellular and humoral immune response reaching the neutralizing antibodies formation, although those antigens are less immunogenic compared to RBD produced in Chinese Hamster Ovary cells [105]. This variation is associated with the three *N*-glycosylation sites (until now, only positions N331 and N343 are experimentally verified) and four O-glycosylation sites (T323, S325, T333, and T345 experimentally tested), which participate in protein conformation, immunogenicity, and antigen presentation [105, 106]. Regardless, in this study, the unglycosylated RBD produced in *E. coli* comprises the non-natively glycosylated apical RBD regions, that are recognized by neutralizing antibodies with high affinity [2, 107, 108]. The RBD and RBD-P produced here were immunogenic and elicited neutralizing antibodies when administrated intramuscularly. Remarkably, this work evoked the improvement of the immunogenicity of non-glycosylated antigens through the oral route, a result that was achieved by adding the 40AV peptide. This is coupled with the fact that bacterial models are widely used in subunit vaccine production due to their simplicity of implementation and substantial cost reduction. Even more, unglycosylated antigens could avoid the auto-antibodies production against host carbohydrates produced in SARS-CoV-2 infection [109]. However, impurities from *E. coli*, like endotoxins (lipopolysaccharides or Lipid A), are responsible for triggering proinflammatory cytokine production, which is important in the design of vaccines administrated parenterally [110, 111]. In this work, this effect was mimicked by the endotoxin synthetic analog S6322 used as an adjuvant during intramuscular administration, while, in oral doses, high concentrations of LPS have been administered in mice without significant effects [112]. Although the elimination of endotoxins was not considered in this work, the amount of these decreases by orders of magnitude due to the purification and according to other bioprocesses [113]. Even more, future analysis of the processes will take care of the removal and characterization of endotoxins and other host contaminants [114].

Our findings highlight the importance of the modified recombinant RBD antigen, providing a basis for the development of an oral SARS-CoV-2 vaccine candidate that induces immune responses. Results spotlight an RBD production bioprocess that can be scalable with the improvement in the immunogenicity of RBD by the fusion peptide in a simple emulsion formulation. Importantly, the peptide-fused RBD in the emulsion generated neutralizing antibodies in immunized mice. Since the vaccine candidate is administered orally, it is necessary to assess IgA isotype immunoglobulins, as well as components of cellular immunity, work that is currently in progress, also further evaluation of the protective capacity in a biological model of SARS-CoV-2. Moreover, determining the antigenic presentation level of the proposed antigen and its ability to induce IgA isotype switch-related cytokines on dendritic cells by flow cytometry and fluorescence microscopy is considered crucial. This work contributes to the knowledge, challenges, and advances for the development of mucosal vaccines for SARS-CoV-2, which is important to generate knowledge between basic, translational, and clinical research.

### Supplementary Information

**Additional file 1**: **Figure S1.** MALDI-TOF mass spectrum for intact RBD and RBD-P with placed on a CHCA (α-Cyano-4-hydroxycinnamic acid) matrix. **Figure S2.** Cross sections of recombinant *E. coli* producing RBD (left) and RBD-P (right), seen under transmission electron microscopy (TEM). **Figure S3.** Murine serum obtained from immunization with the recombinant RBD-P or RBD from Wuhan (x-axis).

## Data Availability

All data generated or analyzed during this study are included in this article and its supplementary files.
