# Supplementary material for: Oral administration of a recombinant modified RBD antigen of SARS-CoV-2 as a possible immunostimulant for the care of COVID-19
Source: Microb Cell Fact. 2024 Feb 6;23:41. doi: 10.1186/s12934-024-02320-5 (PMC10848483; doi:10.1186/s12934-024-02320-5)
Supplement: Supplementary file 1 — Additional file 1: Figure S1. MALDI-TOF mass spectrum for intact RBD and RBD-P with placed on a CHCA (α-Cyano-4-hydroxycinnamic acid) matrix. Figure S2. Cross sections of recombinant E. coli producing RBD (left) and RBD-P (right), seen under transmission electron microscopy (TEM). Figure S3. Murine serum obtained from immunization with the recombinant RBD-P or RBD from Wuhan (x-axis). [file 12934_2024_2320_MOESM1_ESM.docx]

**Oral administration of a recombinant modified RBD antigen of SARS-CoV-2 as a possible immunostimulant for the care of COVID-19**

Norma A. Valdez‑Cruz ^1,2^*, Diego Rosiles-Becerril ^1^, Constanza E. Martínez-Olivares ^1^, Enrique García‑Hernández ^3^, Laura Cobos-Marín ^4^, Daniel Garzón ^5^, Francisco E. López-Salas ^1^, Guadalupe Zavala ^6^, Axel Luviano ^7^, Alejandro Olvera ^8^, Alejandro Alagón ^8^, Octavio T. Ramírez ^8^, Mauricio A. Trujillo‑Roldán ^1,2^*


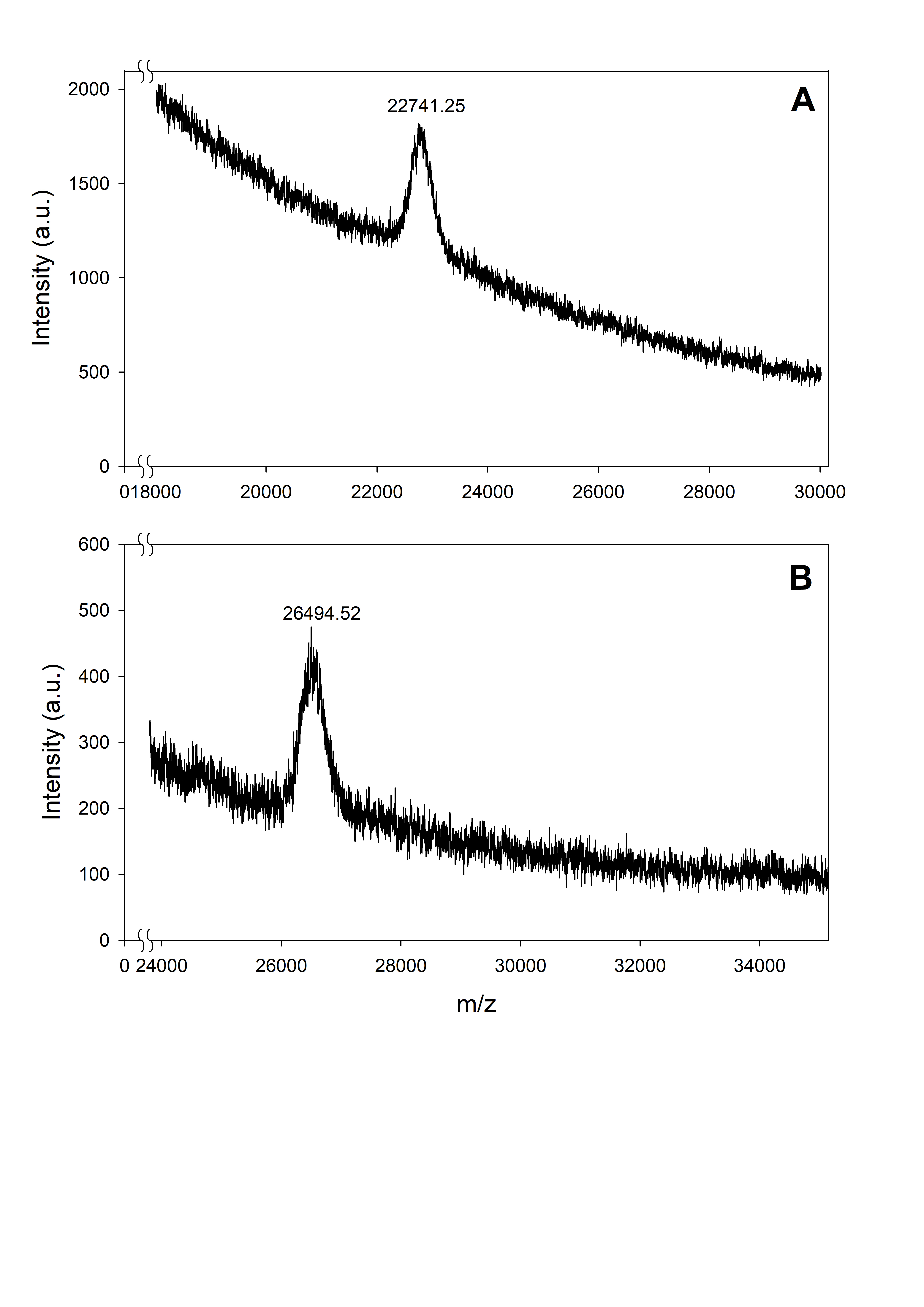


**Figure S1.** MALDI-TOF mass spectrum for intact RBD and RBD-P with placed on a CHCA (α-Cyano-4-hydroxycinnamic acid) matrix. Analysis was performed in a matrix-assisted laser desorption ionization time-of-flight (MALDI-TOF, Bruker Microflex) equipment with a 20-Hz nitrogen laser at I = 337 nm. Spectra were recorded in linear positive mode for the mass range 10,000 to 60,000 Da.


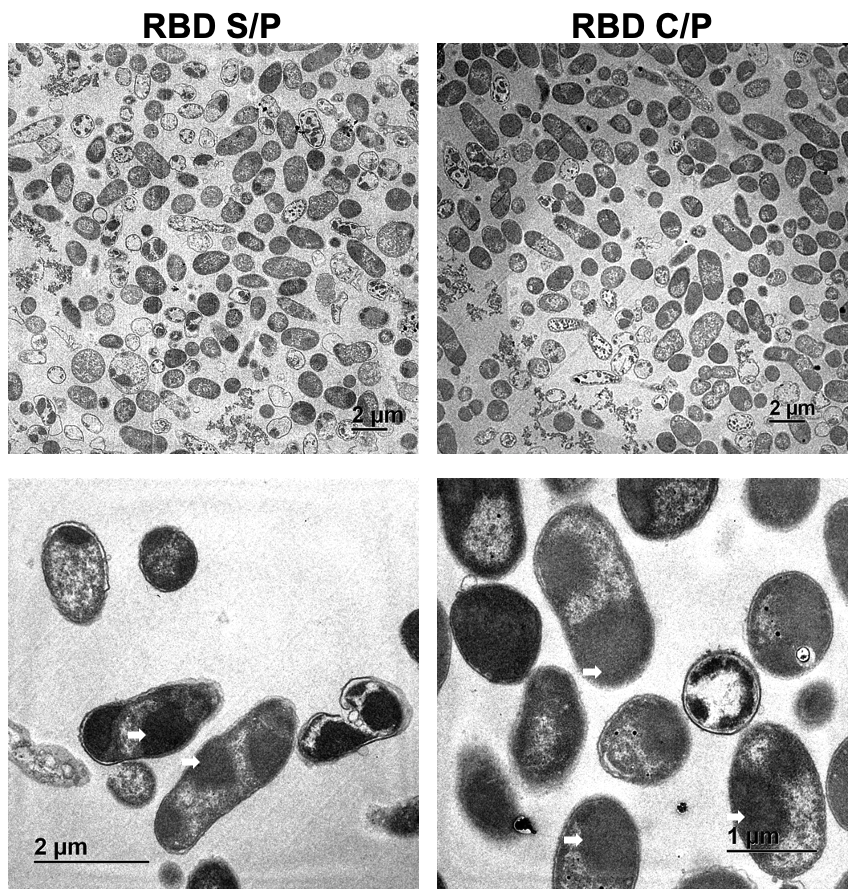


**Figure S2.** Cross sections of recombinant *E. coli* producing RBD (left) and RBD-P (right), seen under transmission electron microscopy (TEM). Cells were harvested 5 h after induction in bioreactors (2.0 and 1.0 μm scale bars). The inclusion bodies are observed as electro-dense bodies inside the cell (some are shown with white arrows).


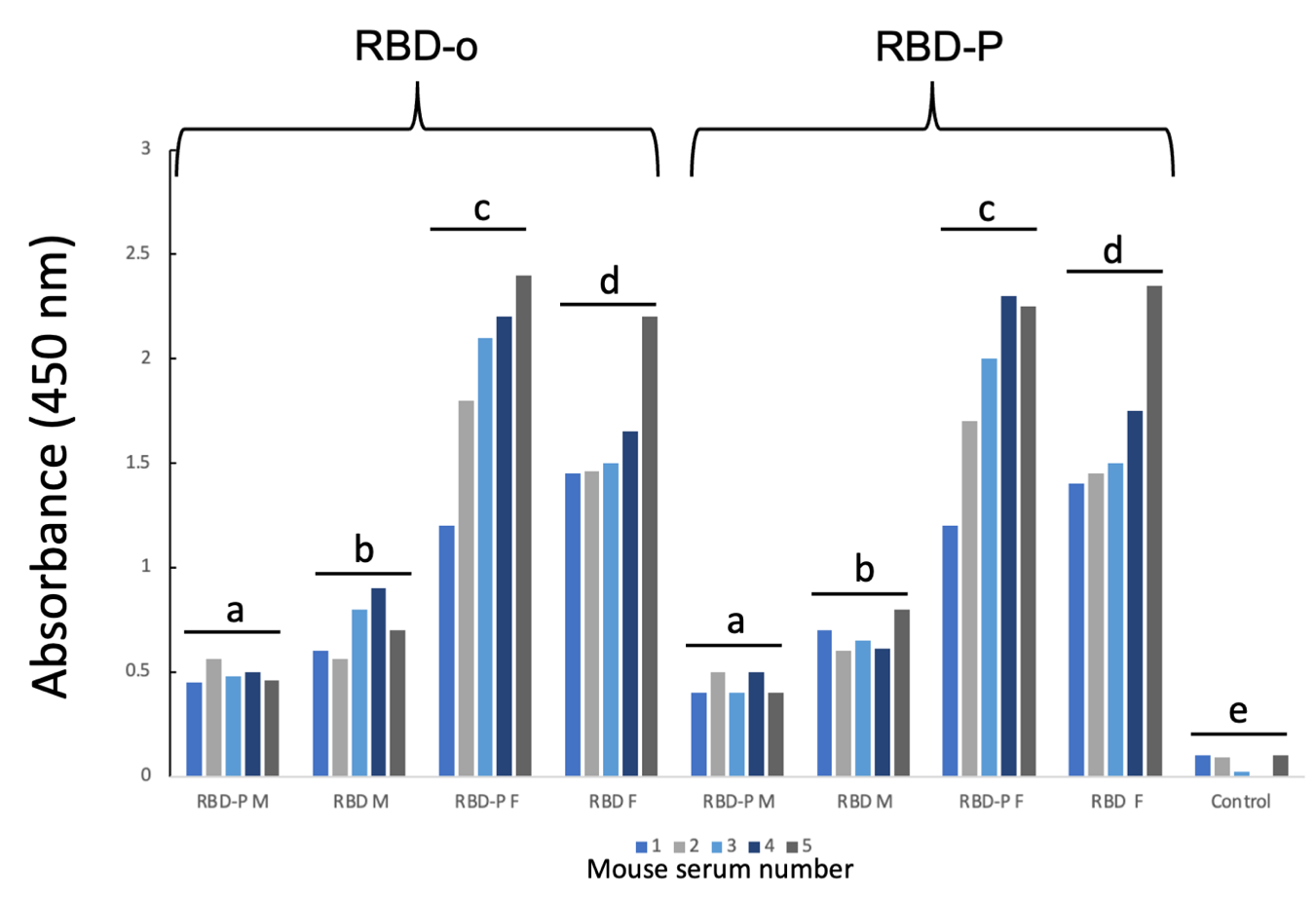


**Figure S3.** Murine serum obtained from immunization with the recombinant RBD-P or RBD from Wuhan (x-axis). The absorbance value measured at 450 nm is presented on the y-axis, (murine serum dilution 1:200), using 0.5 µg of recombinant RBD omicron or RBD-P protein attached. Goat anti-mouse IgG (H+L) antibody (HRP) (Jackson Immunoresearch, USA) dilution 1:5,000 was used as the secondary antibody. For all variables with the same letter, the difference between the means is not statistically significant. If two variables have different letters, they are significantly different (p < 0.05).
